# Single cell multi-omic reference atlases of non-human primate immune tissues reveals CD102 as a biomarker for long-lived plasma cells

**DOI:** 10.1038/s42003-022-04216-9

**Published:** 2022-12-21

**Authors:** Ryan P. Staupe, Kenneth E. Lodge, Nithya Thambi, David Toole, Alex M. Tamburino, Dan Chang, Bonnie J. Howell, Daria J. Hazuda, Kalpit A. Vora, Nicole L. Sullivan

**Affiliations:** 1 Infectious Diseases and Vaccines, MRL, Merck & Co., Inc, West Point, PA USA; 2 LAR, Integrative Vet Medicine, MRL, Merck & Co., Inc., West Point, PA USA; 3 Atlas Data Systems, Berkeley Heights, NJ USA; 4 Large Data Delivery Services, MRL, Merck & Co., Inc, West Point, PA USA; 5 Genome & Biomarker Sciences, MRL, Merck & Co., Inc, West Point, PA USA; 6 Genome & Biomarker Sciences, MRL, Merck & Co., Inc, Cambridge, MA USA; 7 Present Address: AbbVie Inc, Cambridge, MA USA; 8 Present Address: Quantitative Biosciences, MRL, Merck & Co., Inc., West Point, PA USA; 9 Present Address: Vaccine & Medical Affairs, Merck & Co., Inc., West Point, PA USA

**Keywords:** Immunogenetics, Lymphoid tissues

## Abstract

In response to infection or immunization, antibodies are produced that provide protection against re-exposure with the same pathogen. These antibodies can persist at high titers for decades and are maintained by bone marrow-resident long-lived plasma cells (LLPC). However, the durability of antibody responses to immunization varies amongst vaccines. It is unknown what factors contribute to the differential longevity of serum antibody responses and whether heterogeneity in LLPC contributes to this phenomenon. While LLPC differentiation has been studied extensively in mice, little is known about this population in humans or non-human primates (NHP). Here, we use multi-omic single-cell profiling to identify and characterize the LLPC compartment in NHP. We identify LLPC biomarkers including the marker CD102 and show that CD102 in combination with CD31 identifies LLPC in NHP bone marrow. Additionally, we find that CD102 is expressed by LLPC in mouse and humans. These results further our understanding of the LLPC compartment in NHP, identify biomarkers of LLPC, and provide tissue-specific single cell references for future studies.

## Abstract (web summary)

CD102 is an enriched biomarker for long-lived plasma cells in non-human primates (NHPs) and is conserved across NHPs, humans, and mice.

## Introduction

Vaccines are one of the most important public health measures available to control the spread of disease. Protection from vaccines relies on the induction of robust immunity post-immunization and the induction of immunological memory and high-affinity antibodies capable of mediating pathogen clearance and neutralization^1–3^. B cell memory is formed as the result of B cell differentiation and selection and is split into a cellular component, memory B cells (MBC) and plasma cells (PC), and a humoral component, secreted antibody^4–6^. Maintenance of serum antibody titers is accomplished by the continual production of antibodies by long-lived PC (LLPC) which reside in the bone marrow (BM) and gut associated lymphoid tissues (GALT)^7–10^. Serum antibody titers are used as a reliable biomarker for durability of the immune response and threshold of immune protection^1–3^. Balancing the output of memory MBC and PC during an immune response is key to maintaining long-term immunological protection post-vaccination.

While many vaccines induce antibody responses that persist for decades, a subset of vaccines, like the TDAP vaccine or current SARS-CoV2 mRNA vaccines, have been found to induce antibody responses that persist for substantially less time^11–14^. It is currently unclear what factors contribute to the differential durability of antibody responses to these vaccines. Antigen valency, duration of antigen availability (replicating vs. non-replicating), choice of adjuvant, and vaccine platform can all impact the quality and quantity of vaccine-induced immunological memory^15–18^. As the primary source of vaccine-induced serum antibody, understanding how LLPC are formed and sustained in the BM in response to vaccination is critical for the development of high-quality durable vaccines. Indeed, vaccines that induce more durable antibody responses also induce higher numbers of BM-resident LLPC^8,19,20^. A similar correlation between serum antibody titers and circulating MBC has been observed for select vaccine antigens but not all, further highlighting the importance of the LLPC compartment in determining vaccine durability^11^.While differences in the total number of LLPCs could explain the differences in vaccine-induced antibody durability, the BM LLPC compartment is known to be heterogenous, comprised of both shorter- and longer-lived cells with unique cellular phenotypes^21^.Whether and how cellular heterogeneity within the LLPC contributes to vaccine durability is currently unclear.

PC are typically defined by the expression of CD138, low or absent expression of B-cell lineage markers CD19 and CD20, and their spontaneous secretion of antibody^22^. Additionally, PC express high levels of IRF4, BLIMP1, and XBP1 which are necessary to maintain the PC transcriptional program^22,23^.The current dogma states that the majority of PC, which are generated in secondary lymphoid organs after B-cell activation, have a very short lifespan but a subset of the PC generated can traffic to the BM and GALT where they establish residence^10,24^. LLPC are relatively rare within the BM and typically make up <1% of total cells. Surface protein expression of CD38^9^, CXCR4^25^, CD27^26^, and Sca1^27^ have all been used alone or in combination with CD138 to identify plasma cells in mice and humans; however, heterogeneity in the expression of these and other protein markers within the LLPC population has been observed^21,26–29^. While previous studies have profiled the transcriptomic^28^, epigenetic^30,31^, and metabolomic^32^ regulators of LLPC differentiation in mice, the bone marrow residence and rarity of LLPC has made deep molecular profiling of these cells challenging in humans^9,33^. Overcoming these limitations will be key to understanding the generation of LLPC and could enable the development of more durable vaccines.

Non-human primates (NHP) share many behavioral, genetic, and immunologic features with humans and are an essential model in biomedical research for understanding human vaccine responses^34,35^. Immune responses in NHP mirror many of the differentiation pathways of the human immune system including the generation of LLPC and their subsequent trafficking to the BM in response to vaccination^8,36,37^. Importantly, in contrast to humans, NHP models are more easily amenable and immune tissues from NHP are more easily accessible. Further, NHP models reflect human immune responses better than those of mice and represent a larger source of biomaterial for deep profiling efforts of rare cell populations like LLPC. Similarly, to mice and humans, LLPC in NHP express CD138 and lack expression of major immune cell lineage markers. Identification of LLPC in NHP is often challenging due to unreliability of protein staining for CD138 by flow cytometry and loss of CD138 expression following cryopreservation^37,38^. Therefore, other markers are needed to identify LLPC in NHP. CD49d, CD31, and CD98 expression have previously been described as NHP LLPC markers but a comprehensive assessment of LLPC markers in NHP has not been completed^37^. Many challenges remain to the study of LLPC in non-human primates including the availability of reagents to identify proteins and genes of interest. Yet, despite these challenges, non-human primates are still an attractive model system for understanding the biology of LLPC at steady state and in response to vaccination.

Here, we report a two-pronged approach to characterizing LLPC in NHP. First, using high-throughput flow cytometry, we screen 361 commercially available anti-human antibody clones for cross-reactivity with rhesus macaque. We identify 112 antibody clones that show cross-reactivity with rhesus cells and investigate the expression of these markers on lymphocytes in the blood, lymph node (LN), and BM. Second, using droplet-based single-cell transcriptome and surface protein profiling, we report the creation of multi-modal single-cell reference atlases of rhesus peripheral blood mononuclear cells (PBMC), LN, and BM comprising over 200,000 cells. Using these atlases, we investigate the plasma cell compartment in rhesus macaques and identify CD102 as an enriched biomarker for this population. Further, we show that LLPC expression of CD102 is conserved across rhesus macaque, humans, and mice and allows for functional enrichment of antibody-secreting cells in rhesus and humans. In addition, our multi-modal rhesus immune tissue atlases improve our in-depth understanding of the cellular composition of rhesus tissues, providing a valuable reference for future studies, in the process delineating key markers of LLPC in NHP.

## Results

### High-throughput antibody screen identifies antibody clones cross-reactive between human and rhesus macaque

**Fig. 1:**
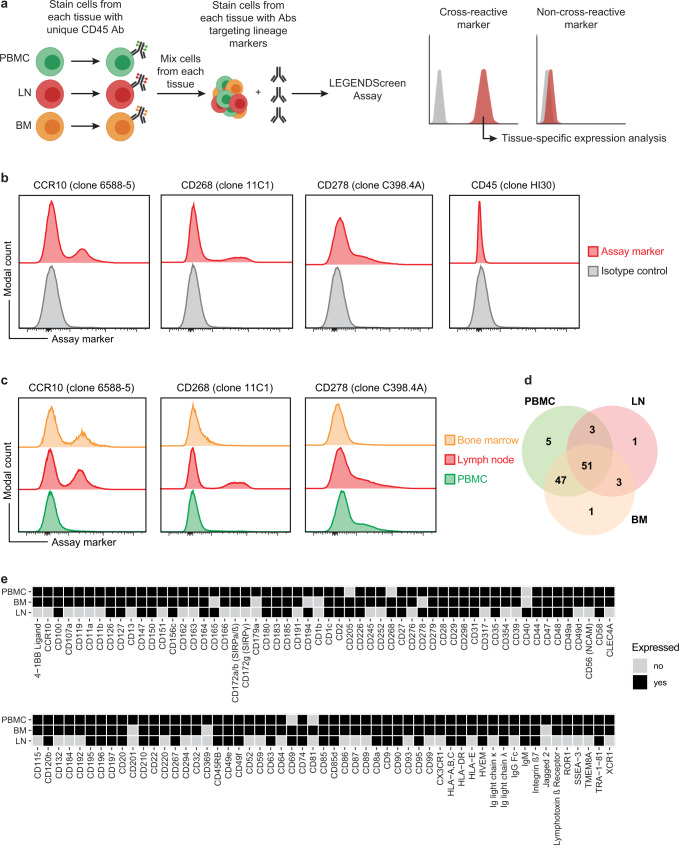
Identification of human-rhesus cross-reactive antibodies. **a** Assay scheme for multiplexing cells from different tissues prior to input into high-throughput flow cytometry-based antibody screen (LEGENDScreen). Briefly, cells from each tissue are labeled with a unique CD45-fluorophore combination prior to being mixed and stained with a panel of antibodies against lineage markers. Cells from each tissue are demultiplexed during analysis (right panel). **b** Histograms of indicated marker expression on lymphocytes for LEGENDScreen assay. Assay marker is in red and isotype control is in gray. **c** Histograms of tissue-specific expression patterns of indicated markers. Cells from bone marrow, lymph node, and PBMC are colored orange, red, and green, respectively. **d** Venn Diagram depicting shared and unique expression patterns of the 112 human-rhesus cross-reactive antibodies identified. Overlaps between circles indicate shared expression between tissue compartments. **e** Heatmap displaying expression patterns of the 112 human-rhesus cross-reactive antibodies identified across the tissues compartments assessed.

LLPC are terminally differentiated cells which are produced during an immune response to antigenic stimulus by responding B cells^4,22^. LLPC reside in the BM where they persist for decades secreting antibody into the blood^7,9^ and are traditionally defined by the co-expression of CD138 (*SDC1*), Blimp1 (*Prdm1*), and in humans CD38^22^. CD138 has also been used to define LLPC in NHP but in our hands staining with commonly available anti-CD138 antibodies has been unreliable for detecting this population consistently by flow cytometry (Supplementary Fig. 1a, b)^37^. CD138 is also expressed on hematopoietic progenitors in the BM and additional markers with or without CD138 may add confidence in identifying LLPC within this tissue compartment^39^. Additionally, while resources exist that report antibody clones that are cross-reactive between NHP and humans, the markers tested are limited and typically only PBMC are assessed^40,41^. Therefore, we set out to define new markers for LLPC in NHP. We used a two-pronged approach towards marker discovery employing both a semi-biased (flow cytometry with known antibodies) and unbiased method (multi-modal single-cell profiling) with the semi-biased method informing our unbiased method. For the semi-biased method, we used a high-throughput flow cytometry assay that allowed assessment of anti-human antibodies targeting 361 individual surface proteins (Fig. 1a). Due to the cellular yields needed to perform this assay, and the low frequency of LLPC in the BM, and the BM-residency of LLPC we choose to use isolated single-cell suspensions from NHP BM, PBMC, and LN as inputs into the assay. Cells from each of these tissues were individually labeled with a unique anti-CD45-fluorophore combination that enabled demultiplexing of cells from each tissue after acquisition and during analysis (Fig. 1a). We reasoned that this approach would allow us to define anti-human antibody clones that cross-react with their NHP counterpart defined as the semi-biased approach of this experiment.

Using this approach, we were able to identify anti-human antibody clones that cross-reacted with NHP surface proteins (Fig. 1b and Supplementary Data 1) and were able to identify distinct patterns of expression across CD45 + cells within the PBMC, LN, and BM (Fig. 1c). We observed binding of known cross-reactive clones for CCR10 (clone 6588-5), CD268 (clone 11C1), and CD278 (clone C398.4 A) (Fig. 1b). Binding of anti-CD45 (clone H130) which is known to not be cross-reactive between human and NHP was not observed^40,41^. Expression of CCR10 was observed in lymphocytes from the BM and LN but not the PBMC (Fig. 1c). CD268 was expressed primarily on lymphocytes in the LN and on a small subset of cells within the BM. Expression of CD278 was observed primarily on cells from the LN and PBMC but not the BM. A total of 112 antibodies were determined to be cross-reactive between human and NHP with many showing distinct tissue expression patterns (Fig. 1d, e and Supplementary Data 1). Overall, these data identified antibody clones that are cross-reactive between human and NHP and provide insight into the tissue-specific expression of the markers assessed. However, while these data are an important resource, the cutoffs used to call a marker positive may preclude the inclusion of markers expressed on smaller populations of cells like LLPC which comprise ~1% of the bone marrow lymphocyte population. It is likely that more than 112 of the 361 markers assessed are cross-reactive.

### Creation of single-cell multi-modal atlases of rhesus PBMC, LN, and BM

**Fig. 2:**
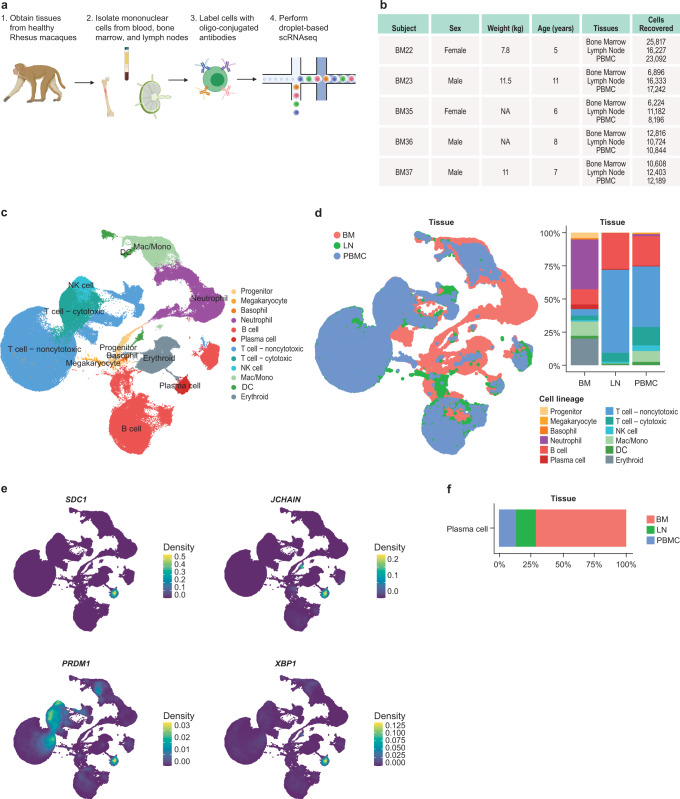
Multi-modal single-cell atlas of rhesus macaque immune tissues. **a** Experimental design for rhesus single-cell data generation. Tissues were collected from healthy rhesus macaques. Single-cell suspensions were isolated from fresh blood, lymph nodes, and bone marrow. To enable profiling of cell surface proteins, cells were labeled with a panel of 124 oligo-conjugated antibodies before being subjected to droplet-based single-cell RNAseq on the 10x Chromium platform. Diagram created with BioRender.com. **b** Table of animal subject metadata for NHP included in single-cell multi-omic analysis and number of cells recovered across tissue compartments assessed. NA indicates data not available. **c** UMAP visualization of cells derived from NHP BM, LN, and PBMC. Cells are colored by major cell lineage. **d** Left panel: UMAP visualization of cells by tissue of origin. Right panel: Distribution of major cell lineages by tissue. **e** Expression of canonical PC genes at the RNA level. Expression is visualized as the kernel density estimate for each RNA. **f** Proportions of PC across the three tissues profiled.

We next took an unbiased approach towards identifying markers of LLPC in rhesus macaques. To identify LLPC in an unbiased fashion, we performed multi-modal droplet-based single-cell transcriptome and surface proteome profiling on cells from NHP BM, PBMC, and LN (Fig. 2a)^42,43^. To enable profiling of the surface proteome of these cells, we cross-referenced the human-NHP cross-reactive antibody markers we identified in Fig. 1, published databases, and commercially available oligo-conjugated reagents to come up with a list of antibodies (115 surface proteins and 9 isotype controls) to be included in the panel (Supplementary Data 2). We targeted 20,000 cells from each of the PBMC, LN, and BM from five individual NHP (Fig. 2b). After stringent quality control and filtering, we identified a total of 201,773 cells with 62,493, 67,226, and 72,054 cells derived from the BM, LN, and PBMC, respectively. Unbiased clustering identified 55 clusters of cells which could be broadly grouped into 12 main hematopoietic cell lineages by their expression of canonical marker genes and surface proteins (Fig. 2c, Supplementary Fig. 2a, b and Supplementary Data 3). Samples from each animal were evenly split across all the identified clusters and cell lineages (Supplementary Fig. 2c). Similarly, after batch correction, clustering of cells was not biased by other biological or technical covariates such as sex (Supplementary Fig. 2d). Consistent with observations in humans^44–46^ and mice^47,48^, the BM was comprised primarily of progenitor cells, maturing monocytes and granulocytes, developing B cells, and erythroid precursors whereas the LN and PBMC were comprised primarily of mature B and T cells (Fig. 2d and Supplementary Fig. 2e). Importantly, we were able to identify a cluster of 3,040 PC by their high expression of *SDC1, JCHAIN, PRDM1*, and *XBP1* (Fig. 2e). The PC cluster consisted of ~1.5% of the total cells in the dataset. As expected, given the bone marrow residence of LLPCs, 71.1% of the cells within the PC cluster were derived from the BM with the remaining percentage being split between cells from the LN (15.7%) and PBMC (13.2%) (Fig. 2f).

### Identification of enriched markers for BM LLPC in NHP

**Fig. 3:**
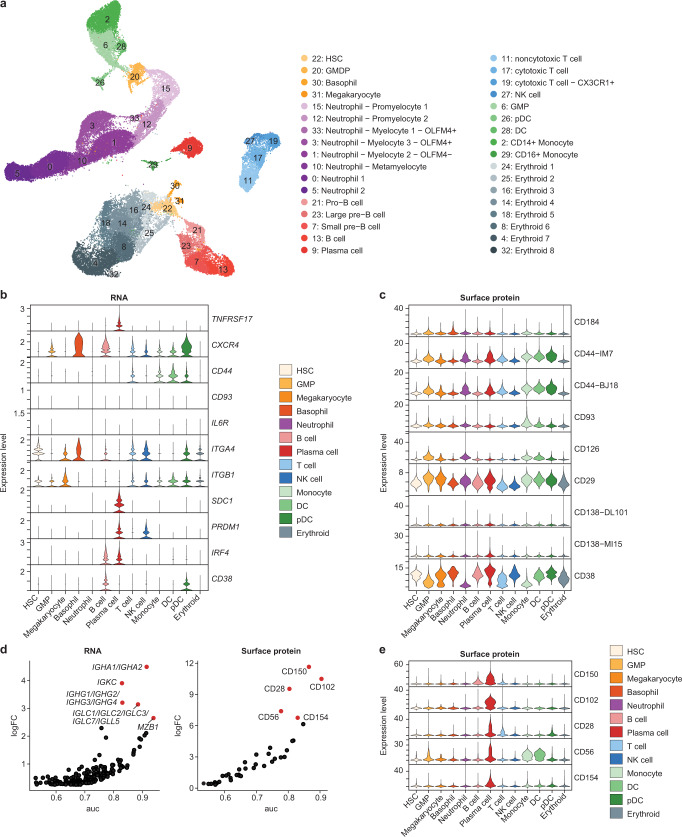
Identification of enriched markers for BM LLPC in NHP. **a** UMAP visualization of cells from NHP BM depicting results of unbiased clustering and cell type annotation. **b** mRNA expression in LLPC in NHP of previously identified LLPC markers in mice and humans. **c** Expression of surface protein markers on LLPC in NHP of previously identified LLPC markers in mice and humans. **d** Differential gene expression analysis for LLPC in NHP compared to all other cells isolated. Genes are ranked by expression (log fold-change; logFC) and classification power (AUROC; auc). Top 5 genes are labeled. Ensembl gene identifiers were changed to closest human homolog for the following RNA transcripts to enable ease of interpretation: ENSMMUG00000002764: human homolog IGHA1/IGHA2, ENSMMUG00000015202: human homolog IGHG1/IGHG2/IGHG3/IGHG4, ENSMMUG00000044861: human homolog IGLC1/IGLC2/IGLC3/IGLC7/IGLL5. **e** Differential surface protein marker expression analysis for PC in NHP compared to all other cells isolated. Surface protein markers are ranked by expression (log fold-change; logFC) and classification power (AUROC; auc). Top 5 markers are labeled.

Having identified PC in our single-cell data, we next sought to understand the RNA and surface protein markers that distinguish them from the rest of the cells in the dataset. For this analysis, we focused on the BM, as this is the primary site of LLPC residence^7–9^ and 70% of the PC we observed within out single-cell data were derived from the BM (Fig. 2f). Unbiased clustering of BM-derived cells identified 32 unique clusters of cells (Fig. 3a). Annotation of these clusters using both RNA (Supplementary Fig. 3a, b and Supplementary Data 4) and surface protein information (Supplementary Fig. 3c, d and Supplementary Data 4) identified hematopoietic progenitor and mature lymphocyte populations consistent with previously identified hematopoietic cell populations identified in human and mouse BM^44,49,50^. This included clusters of cells undergoing major steps in neutrophil granulopoiesis (clusters 15, 12, 33, 3, 1, 10, 0, and 5) and B cell development (clusters 21, 23, 7, and 13) (Fig. 3a)^51,52^. Further, we were able to identify a distinct cluster of LLPC (cluster 9) that had high expression of *SDC1*, *JCHAIN*, *PRDM1*, and *XBP1* (Supplementary Fig. 3b). Interestingly, while we observed surface protein expression of CD138 on the LLPC cluster, binding of anti-CD138 clone DL101 was also observed in the developing neutrophil clusters whereas binding of anti-CD138 clone MI15 was restricted to the LLPC cluster (Supplementary Fig. 3c). Together, these results identify LLPC within NHP BM and demonstrate that, while CD138 is indeed a marker of LLPC within NHP BM, choice of antibody clone is critical for precise identification of this population.

While the focus of these studies are LLPC, we provide additional tissue atlases with lineage and fine cell type annotations for NHP PBMC and LN (Supplementary Figs. 4, 5). Additionally, we provide RNA and surface protein markers for each of the 141 unique clusters identified across the four datasets (Supplementary Data 3–6). Datasets were manually annotated by investigation of canonical marker genes and surface proteins for major cell lineages and differentially expressed cluster marker genes and proteins. These references provide many insights into the composition of the NHP immune system and will be valuable resources for future studies.

We next sought to understand if LLPC from NHP expressed known PC markers identified in mice or humans. Initially, we assessed if NHP LLPC expressed BCMA, CXCR4, CD44, CD93, IL6R, VLA-4, CD138, CD38, and CD27 which have all been previously identified as being expressed by LLPC in mice and humans^22^. Expression of *BCMA*, *CXCR4*, the two subunits of VLA-4 (*ITGA4* and *ITGB1*), and *SDC1* which encodes for CD138 were observed at the RNA level within the LLPC cluster (Fig. 3b). Expression of *CD44*, *IL6R*, *CD93*, and *CD38* at the RNA level were either lowly expressed or not expressed at all on LLPC in NHP. From this data we cannot rule out that inefficiencies in RNA capture for droplet-based single-cell transcriptomics accounting for the lack of expression of some of these markers. However, transcripts for *CD44* and *CD38* were observed in other cell lineages suggesting true lack of LLPC expression for these markers at the RNA level. Interestingly, while we didn’t observe expression of *CD44* and *CD38* at the RNA level, both these markers were expressed on the cell surface at the protein level (Fig. 3c). These results demonstrate that LLPC from NHP share many known markers with LLPC in mice and humans.

Differential expression analysis identified 129 RNA markers (Fig. 3b) and 41 protein markers (Fig. 3c) as upregulated on PC compared to all other cells (Supplementary Data 4). We ranked genes and surface protein markers based on their differential expression (log fold-change) and classification power (AUROC). At the RNA level and as expected, immunoglobulin genes *ENSMMUG00000002764* (human ortholog *IGHA1* and *IGHA2*), *IGKC*, *ENSMMUG00000044861* (human orthologs *IGLC* genes), and *ENSMMUG00000015202* (human orthologs *IGHG1-4*) were among the highest differentially expressed and classifying genes for NHP LLPC (Fig. 3d). Similarly, genes related to the ER stress response (*HERPUD1*) and protein folding (*FKBP1, SSR4*) were also highly expressed in the LLPC cluster consistent with their antibody-secreting function (Supplementary Data 4). CD150, CD102, CD28, CD56, and CD154 were among the highest differentially expressed surface proteins on LLPC (Fig. 3d). Broadly, the differentially expressed surface proteins were markers of cell signaling such as cytokine receptors or cellular adhesion markers. CD150^53^, CD56^54^, CD28^55^ are known to be expressed on normal or malignant PC; however, CD102 and CD154 are not known to be expressed on LLPC. Surface protein expression of CD31 and CD49d were also observed on the BM-resident LLPC population, consistent with previous studies of BM-resident LLPC in NHP (Supplementary Data 4)^37^. Of the surface protein markers enriched within the LLPC population, CD102 expression was highly restricted to LLPC whereas expression of many of the other top markers was observed by other cell types within the BM (Fig. 3e). These results suggest that CD102 alone or in combination with other markers could enable functional enrichment of LLPC from NHP BM. Overall, unbiased identification of LLPC in our single-cell data and multi-modal differential expression analysis identified known and novel enriched markers for LLPC in NHP.

### CD102 in combination with CD31 enables enrichment of antibody-secreting cells independent of CD138

**Fig. 4:**
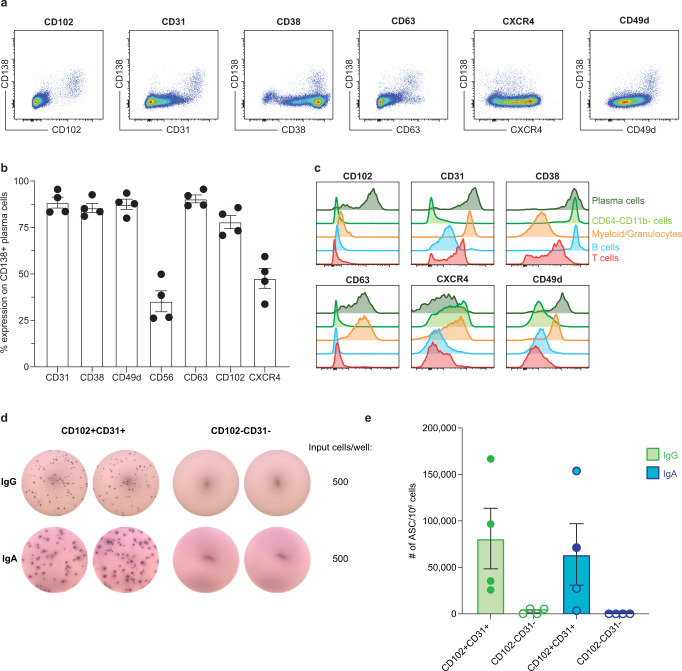
CD102 and CD31 enable enrichment of antibody-secreting cells independent of CD138. **a** Representative flow plots of marker expression for CD102, CD31, CD38, CD63, CXCR4, and CD49d. Cells were previously gated as CD3-CD20-CD64-CDllb- single lymphocytes. **b** Quantification of marker expression on CD138+ plasma cells in rhesus macaque bone marrow; *n* = 4 biologically independent animals. **c** Representative histograms of indicated marker expression for various immune cell lineages identified in the bone marrow of rhesus macaques. Dark Green = plasma cells (CD138 + CD3−CD20−CD64−CDllb− single lymphocytes), light green = non-B/T (CD3−CD20−) CD64−CD11b− cells, orange = non-B/T (CD3−CD20−) CD64 + CD11b + cells, blue = B cells (CD20 + cells), red = T cells (CD3 + cells). **d** Representative images of ELISPOT wells coated with either anti-IgG or anti-IgA. CD102 + CD31 + or CD102−CD31− cells from rhesus macaque bone marrow were plated in duplicate and serial diluted (2-fold) down the plate. Loadings for the top dilution are indicated. **e** Quantification of total-IgG and total-IgA ELISPOTs for the unsorted, CD102 + CD31 + , and CD102−CD31− populations. Quantification of the number of antibody-secreting cells (ASC) per million input cells. Error bars depict standard error of the mean. *n* = 4 biologically independent animals.

Having identified CD150, CD102, CD56, CD31, CD63, and CD154 as markers of LLPC within NHP BM in our single-cell datasets, we next sought to validate the expression of these markers using another independent method. Using flow cytometry, we stained cells and measured the expression of a subset of markers identified in the single-cell analysis. We identified LLPC as CD3−CD20−CD64−CD11b− and CD138 + . Using this gating strategy, we measured expression of CD102, CD31, CD38, CD63, CXCR4, CD49d on BM-derived LLPCs (Fig. 4a). When co-expression of CD138 and the marker of interest were visualized, we observed clear separation of a population of cells for most markers assessed. We next determined the fraction of LLPCs expressing the markers of interest. CD31, CD38, CD49d, CD63, and CD102 were expressed on nearly (~80%) of all CD138 + plasma cells (Fig. 4b). CD56 and CXCR4 were expressed on less than 50% of the cells consistent with our single-cell data (Supplementary Data 4). We next wanted to determine how well these markers could distinguish LLPC from the rest of the cells in the BM. CD102 expression was highly restricted to the BM LLPC population whereas CD31, CD38, CD63, CXCR4, and CD49d were highly expressed on other cell populations, in particular myeloid and granulocyte lineage cells (Fig. 4c). Together, these data identify CD102 as an enriched marker for LLPC in NHP BM.

We next sought to confirm our observations that CD102 was indeed a marker for LLPC in NHP BM. To test this, we designed a sorting strategy to purify LLPC from the BM using CD102 and used the sorted cells as input into a total-IgG and IgA ELISPOT assay to measure secreted antibody. In this sorting strategy, we identify putative PC as CD45 + CD3−CD20−CD64−CD11b− cells that co-express CD102 and CD31 (Supplementary Fig. 6a). CD31 was included as a marker along with CD102 because we noticed during our flow validation experiments that gating on CD45 + CD102 + CD31 + cells identified almost 70% of the traditionally gated PC population and this marker is known to be expressed on LLPC in NHP (Supplementary Fig. 6b)^37^. Further, we reasoned that the combination of CD31 and CD102 could enable identification of LLPC independent of CD138 (Supplementary Fig. 6c, d). Using this strategy, we were able to identify antibody-secreting cells in the sorted CD102 + CD31 + fraction but not the CD102−CD31− sorted fraction (Fig. 4d, e). Overall, these results show that LLPC in NHP express CD102 and CD31 and that these markers in combination are able identify antibody-secreting cells isolated from NHP BM.

### CD102 expression is conserved across species

**Fig. 5:**
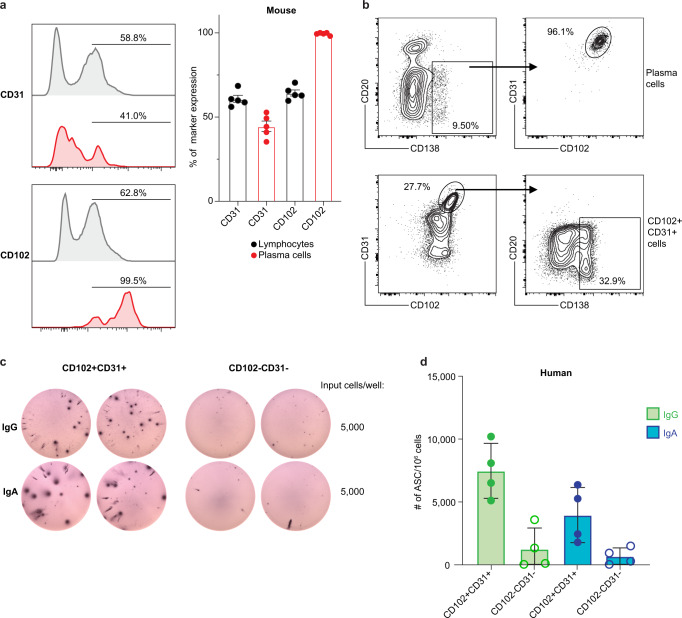
CD102 and CD31 expression is conserved across species. **a** Left panel: Representative histograms of CD31 and CD102 expression on bulk lymphocytes or CD138 + plasma cells on cells from mouse bone marrow. CD138 + plasma cells were previously gated as live, singlets, CD4−CD8−F4/80−NK1.1−IgD−. Lymphocytes were previously gated as live singlets. Right panel: Quantification of CD31 and CD102 expression on bulk lymphocytes or CD138 + plasma cells on cells from mouse bone marrow. Error bars represent standard error of the mean. *n* = 5 biologically independent animals. **b** Top panel: Representative flow plot of plasma cell gates and CD102 and CD31 expression on plasma cells from human bone marrow cells. Bottom panel: Representative flow plot of frequency of plasma cells within CD102 + CD31 + cells. **c** Representative images of ELISPOT wells coated with either anti-IgG or anti-IgA. CD102 + CD31 + or CD102−CD31− cells from human bone marrow were plated in duplicate and serial diluted (2-fold) down the plate. Loadings for the top dilution are indicated. **d** Quantification of ELISPOT results depicting the number of ASCs identified per million input cells for both total-IgG and total-IgA secreting cells. Error bars represent standard deviation from the mean. *n* = 4 biologically independent human subjects.

Given that we identified CD102 as a marker for LLPC in the BM of NHP, we next wanted to determine whether CD102 expression on LLPC is conserved across species. To investigate this, we immunized a cohort of mice intramuscularly (IM) with 50 µg of the hapten 4-Hydroxy-3-nitrophenylacetyl-Chicken Gamma Globulin (NP-CGG). We waited 14 days post-immunization then assessed the expression of CD102 and CD31 on cells from the BM of these mice by flow cytometry. Approximately 60% of bulk lymphocytes expressed CD102 and a similar percentage expressed CD31 (Fig. 5a). In contrast, only 40% of LLPC expressed CD31 whereas nearly 100% of the LLPC expressed CD102. Given that LLPC in the BM of these mice comprise less than 1% of the total lymphocyte populations, these results indicate that nearly all LLPC in mice express CD102, however, the expression of CD102 is not restricted to the LLPC population in mice.

We next sought to determine whether LLPC in humans express CD31 or CD102. We prepared single-cell suspensions from cells isolated from human iliac crest BM aspirates and assessed the expression of CD102 and CD31 on LLPC. We identified LLPC in humans as lymphocytes that were CD45 + CD20lowCD138 + (Fig. 5b). We detected a sizable population of LLPC across samples from four donors and LLPC represented between 5-7% of the total lymphocytes. Like the results seen in NHP, we observed CD102 and CD31 expression on LLPC from human bone marrow with strong co-expression of these markers also observed (Fig. 5b). However, unlike our observations in NHP, where CD102 + expression was restricted to LLPC, CD102 expression was observed on other cells in the BM including CD20low cells. To confirm that CD102 and CD31 were indeed markers expressed on LLPC in human BM, we sorted CD45 + CD102 + CD31 + and CD45 + CD102−CD31− cells and used them as input into total-IgG and total-IgA ELISPOT assays (Supplementary Fig. 7a–c). We observed antibody-secreting cells in the sorted CD102 + CD31 + population but not in the CD102−CD31− population (Fig. 5c, d). Taken together, these results indicate that indeed, CD102 is expressed by LLPC in mouse and human BM. However, unlike in NHP, CD102 expression is not restricted to the LLPC compartment in mice and humans.

## Discussion

Here, we take a two-pronged approach to interrogate LLPC biology in NHP. First, to broaden the list of available reagents for studying the NHP immunity, we conducted a biased screen of human antibody clones targeting cell surface proteins. We identified cross-reactive antibody clones and investigated the expression of these markers on lymphocytes in the PBMC, LN, and BM of NHP. An important strength of this work is that all cells from the tissues profiled were freshly isolated and never cryo-preserved. This provides an advantage because the expression of some cell surface proteins is altered upon freeze-thaw following cryopreservation^37,56^. It is currently unclear whether loss of expression of a particular marker after cryopreservation is due to true loss of the epitope targeted or selective loss of populations of cells. In our experience, this is a particular concern with studying LLPC as we have observed loss of both functional antibody-secreting cells as measured by ELISPOT and expression of CD138 following cryopreservation of NHP BM mononuclear cells.

Second, to identify LLPCs in an unbiased fashion, we performed single-cell transcriptional and surface protein profiling. Advances in multi-omic single-cell profiling enable assessment of thousands of unique genes and hundreds of surface proteins on tens of thousands of single cells in a high-throughput manner^42,43^. Using state of the art techniques, we created reference atlases for three NHP immune tissues. By profiling mononuclear cells from PBMC, LN, and BM we identified PC by their unique transcriptome. Consistent with data from human and mouse, we found that PC are relatively low abundance in the BM and even rarer in the LN and PBMC at baseline. In total, we were able to identify 3040 PC in our data out of over 200,000 individual cells profiled. Importantly, profiling this many cells enabled us to perform truly unbiased marker discovery for LLPC in NHP without biasing our readout using enrichment strategies based on the expression of known PC markers like CD138.

Using these two methods we identified markers for LLPC in NHP, including the novel enriched marker CD102. CD102 is encoded by intercellular adhesion marker 2 (*ICAM2*) and is involved in cell trafficking and adhesion through binding its ligand lymphocyte function-associated antigen 1 (LFA-1). The role of ICAM2 and its closely related family member ICAM1 in regulating B cell biology is poorly understood^57–59^. Previous studies have shown that genetic deletion of ICAM1 and ICAM2 lead to shorter B cell interaction with T cells in the germinal center resulting in impaired affinity maturation of the antibody response, poor recruitment into the germinal center of activated B cells, and critically, impaired induction of PC responses^57^. Further, combined blockade of ICAM1 and ICAM2 results in a reversible depletion of antigen-specific PC from the bone marrow of mice^60^. Together with our results, these findings suggest that ICAM2 may play a role in keeping LLPCs within the BM in NHP. This is further supported by our observations that many of the surface protein markers upregulated on LLPC also had known functions in mediating cell-cell interactions or cellular trafficking. Additionally, our reanalysis of single-cell profiling data in another NHP species, the crab-eating macaque, found that CD102 is specifically and highly expressed on BM LLPC further highlighting a potential role for CD102 in regulating LLPC biology across NHP species^61^. Given that LLPCs within the BM are thought to reside in a cellular niche that supports their quiescent survival over a long period of time^22,62^, the upregulation of cell adhesion and trafficking markers suggests that the homing of LLPC to a particular location and their interaction with specific cells within the BM is critical to their survival and continued function. Further investigation is needed to identify whether LLPC within the BM inhabit a particular cellular niche and which cells within that niche support the longevity of LLPC.

Despite the specific and high expression of CD102 on BM LLPC in NHP, CD102 expression was not specific to the LLPC compartment in mice or humans. We observed that although all LLPC in mouse and human BM express CD102, not all CD102 expressing cells are LLPC. Despite this, CD102 and CD31 in combination with CD138 leads to better, unambiguous identification of LLPC in all species. Further, this raises an intriguing possibility that targeting CD102 or other cellular adhesion markers in humans could provide therapeutic efficacy for treating human disease driven by abnormal PC responses. Multiple myeloma, a PC cancer, and lupus, an autoimmune disorder driven by self-reactive antibody, are two diseases that could benefit from new therapeutic modalities^63^. Multiple myeloma is typically treated with chemotherapy, radiation, or stem cell transplantation^64^. Newer therapies target markers on PC either by monoclonal antibodies targeting CD38^65^ or by cellular therapy targeting B-cell maturation antigen (BCMA)^66^. While these therapies have shown promise, none are curative. One potential reason is the protected localization of PC in the BM. Similarly, autoreactive antibody-secreting PC that are generated during lupus also reside in the BM^67^. Targeting CD102 alone or in combination with other PC adhesion markers could potentiate release of LLPC from their BM niche and lead to PC trafficking into the periphery where they could be more easily targeted for elimination.

In addition to the above PC neoplasms, effective and durable protection following vaccination relies on the generation of immune memory in the form of MBC and LLPC^1–3^. While all commercially available vaccines induce robust immunity, the durability of the antibody responses varies between individual vaccines^11,12,14^. It is currently unclear what factors influence the differences in antibody durability between vaccines, but it is likely that the quantity and quality of antibody-secreting LLPC formed during vaccination play a role. Studying the biology of LLPC in humans and mice has historically been challenging due to limitations in accessing the site of LLPC residence, the BM, and isolating enough cells for deep molecular profiling due to their relatively low abundance. In this work, we addressed these challenges by studying the LLPC compartment in NHP. The NHP model system has two advantages for studying LLPC biology: (1) NHP immune responses more closely mirror those of humans than mice, and (2) enough biomaterial is available to isolate and characterize LLPCs using high-throughput assays. Our studies add to our knowledge of LLPC biology in NHP and identify a unique marker of LLPC in NHP that could be employed to test different vaccine formulations and images that could generate optimal LLPC.

In summary, our studies employed biased and unbiased biomarker discovery and identify CD102 as an enriched marker for LLPC in NHP. We show that CD102 in combination with other markers can be used to identify LLPC across species. CD102 in combination with CD31 allows more precise identification of BM-resident LLPC in NHP which will enable us to better understand the biology of LLPC within the BM, in particular their generation, longevity, and cellular niche. Vaccine formulations that enhance seeding of the LLPC population, as studied in experimental model systems like NHP, could help increase the durability of immune responses in human. Finally, independent of our identification of CD102 as an enriched LLPC marker in NHP, this work also establishes single-cell atlases for three NHP immune tissues, providing a useful framework for future NHP studies.

## Methods

### Study design—rhesus macaque

All animal studies were carried out by the Research Laboratories of Merck & Co., Inc., Kenilworth, NJ, USA at our West Point, PA, USA and all experiments involving laboratory animals were approved by the Institutional Animal Care and Use Committee (IACUC) of Merck & Co., Inc., Kenilworth, NJ, USA. Healthy adult Indian-origin rhesus macaques of either sex were used for these studies. Details on cohort can be found in Fig. 2b. All animals were treatment-naïve for at least 1 year prior to enrollment in these studies.

### Study design—mice

Six- to eight-week-old female C57BL6N mice were purchased from Charles River Laboratories. All mice were communally housed according to IACUC protocols of Merck & Co., Inc., Kenilworth, NJ, USA. in a specific pathogen-free facility. Mice were acclimated in the housing facility for 1 week prior to inclusion in these studies. For immunization studies, mice were immunized with 50 ug 4-Hydroxy-3-nitrophenylacetyl-Chicken Gamma Globulin (NP-CGG, 10–16 conjugation ratio, Biosearch Technologies) formulated in Adju-Phos adjuvant (Invivogen) intramuscularly (IM).

### Study design—human

Fresh whole bone marrow aspirates from healthy donors were obtained from the Stem Cell and Xenograft Core at the Perelman School of Medicine, University of Pennsylvania. The informed consent to collect and use human donor-derived specimens for research was obtained under the protocol “Normal Donor Human Bone Marrow Donation For Research”, which is approved by the University of Pennsylvania’s Institutional Review Board (IRB protocol #701582).

### Rhesus macaque—PBMC processing

Whole blood was collected into either CPT tubes containing sodium heparin (BD Biosciences) or blood collection tubes containing sodium heparin (BD Biosciences). CPT tubes were spun at 1600 × *g* for 25 min with 3 acceleration and no brake at room temperature to separate cell layers. After centrifugation, ~3 ml of plasma was collected prior to collecting the cell layer. PBMC from each CPT tube were pooled prior to being washed with DPBS and pelleted at 400 × *g*. For blood collection tubes, blood was collected from each tube and pooled prior to being aliquoted into 50 ml conicals and underlaid with a layer of room temperature Histopaque-1077 (Sigma Diagnostics). Samples were centrifuged at 800 × *g* for 15 min with 3 acceleration and no brake at room temperature to separate cell layers. Following centrifugation, plasma was collected prior to harvesting PBMC from each CPT tube were pooled prior to being washed with DPBS and pelleted at 400 × *g*. All PBMC samples were subjected to RBC lysis in ACK Lysis Buffer (Gibco) and strained through a 100 um filter (Corning) prior to final resuspension in cRPMI and cell counting. All samples were processed and stored at room temperature unless otherwise noted.

### Rhesus macaque—lymph node processing

Axial, brachial, mesenteric, and inguinal lymph nodes were collected from rhesus macaques <2 h post-necropsy. All lymph nodes collected were pooled together in cRPMI prior to tissue processing. To isolate cells from lymph nodes, collected lymph nodes were trimmed of excess fat, placed in a small amount of cRPMI within a petri dish, mechanically dissociated using dissection scissors to disrupt the lymph node capsule, then mechanically dissociated using the flat end of the plunger from a 30 ml syringe (BD Biosciences). Isolated cells were harvested from the petri dish before being strained through a 100 um filter and washed 3× with DPBS. Mononuclear cells were isolated by underlaying the isolated cell suspension with a layer of room temperature 96% Ficoll (GE Healthcare) prior to centrifugation at 400 × *g* for 30 min with 3 acceleration and no brake. Following centrifugation, mononuclear cells were harvested and washed with DPBS before RBC lysis was performed in ACK Lysis Buffer (Gibco). Cells were resuspended in cRPMI prior to cell counting. All samples were processed and stored at room temperature unless otherwise noted.

### Rhesus macaque—bone marrow processing

Whole femur bone marrow was collected from rhesus macaques <2 h post-necropsy. Whole femur marrow was harvested into sodium citrate containing tubes (BD Biosciences). Cells were isolated from bone marrow samples by placing sample on top of a 100 um cell strainer filter (Corning) followed by mechanical dissociation using the flat end of a 5 ml syringe (BD Biosciences). Cells were washed with DPBS and then underlaid with room temperature 96% Ficoll (GE Healthcare) prior to centrifugation at 400 × *g* for 30 min with 3 acceleration and no brake. Following centrifugation, mononuclear cells were harvested and washed with DPBS before RBC lysis was performed in ACK Lysis Buffer (Gibco). Cells were resuspended in cRPMI prior to cell counting. All samples were processed and stored at room temperature unless otherwise noted.

### Mouse—bone marrow processing

Bone marrow was harvested from the femur and tibia by isolating the leg bones, snipping the top and bottom of each bone to expose the bone marrow, and flushing the bone marrow with cRPMI using a 5 ml syringe fitted with a G18 needle. Isolated bone marrows were pelleted at 500 × *g* for 10 min prior to resuspension in cRPMI and filtration through a 100 um cell strainer set over a 50 ml conical. Cells that did not flow through the filter were mechanically dissociated using the flat end of the plunger from a 5 ml syringe (BD Biosciences) prior to washing with DPBS. Cells were pelleted by centrifugation at 500 × *g* for 5 min prior to RBC lysis with ACK Lysis Buffer (Gibco). Cells were resuspended in cRPMI prior to cell counting. All samples were processed at room temperature and stored at 4 °C unless otherwise noted.

### Human—bone marrow processing

Fresh iliac crest bone marrow aspirates from healthy human donors were collected at the Perelman School of Medicine, University of Pennsylvania, in heparinized syringes and transported to the lab for further processing within 4 h of sample collection. Cells were isolated from bone marrow aspirate samples by placing sample on top of a 100um cell strainer filter (Corning) followed by mechanical dissociation using the flat end of a 5 ml syringe (BD Biosciences). Cells were washed with DPBS and then underlaid with room temperature 96% Ficoll (GE Healthcare) prior to centrifugation at 400 × *g* for 30 min with 3 acceleration and no brake. Following centrifugation, mononuclear cells were harvested and washed with DPBS before RBC lysis was performed in ACK Lysis Buffer (Gibco). Cells were resuspended in cRPMI prior to cell counting. All samples were processed and stored at room temperature unless otherwise noted.

### Flow cytometry—sample staining

Single-cell suspensions isolated from rhesus macaque, human, or mouse tissues were prepared for flow cytometry by first incubating the cells with the appropriate Fc block (rhesus/human: Human TruStain FcX, Biolegend or mouse: purified rat anti-mouse CD16/CD32, BD Biosciences) in combination with a viability dye (Live/Dead Aqua or Violet, ThermoFisher Scientific). Following the Fc block and viability staining step, panels of fluorescently conjugated antibodies were used to stain markers of interest. Details on markers targeted, antibody clones used, and fluorescent conjugates used can be found in Supplementary Data 2. All cells were stained in a 1:1 mix of FACS buffer (1% FBS 2 mM EDTA in PBS) and Brilliant Violet Stain Buffer (BD Biosciences). Staining for human and rhesus samples was conducted at room temperature. Staining for mouse samples was conducted at 4 °C.

### Flow cytometry—LEGENDScreen

Single-cell suspensions from rhesus macaque tissues were used as input into the LEGENDScreen assay (Biolegend). Cell suspensions were incubated with Fc block (Human TruStain FcX, Biolegend) prior to labeling with fluorescently conjugated antibodies. To enable assessment of assay markers on cell derived from individual tissues, cells from bone marrow, lymph node, and PBMC were each labeled with anti-NHP CD45 (clone D058-1283, BD Biosciences) conjugated to a unique fluorescent tag to enable sample multiplexing and unmixing during analysis. Following labeling with CD45, samples were washed 2× with FACS buffer before being stained with antibodies targeting CD3 (SP34-2, BD Biosciences), CD20 (2H7, Biolegend), and CD138 (DL101, Biolegend). 1 × 10^6^ cells were used as input into each well of the LEGENDScreen assay. Samples were acquired on a Symphony A5 (BD Biosciences) using FACSDiva (BD Biosciences).

### Flow cytometry—analysis

All flow cytometry data analysis was performed in FlowJo (v 10.6.2, BD Biosciences). For the LEGENDScreen assay, markers were called positive if at least 10% of cells had signal greater than that of the 95th percentile of the corresponding isotype control as previously described. Tissue-specific expression patterns were determined by demultiplexing CD45 positive cells according to the staining scheme mentioned above and in Fig. 1a.

### Cell sorting

Single-cell suspensions isolated from whole rhesus macaque bone marrow or human bone marrow aspirates were prepared for cell sorting by first incubating the cells with Fc block (Human TruStain FcX, Biolegend). Following Fc block, cells were washed 1× with DPBS before being stained with a cocktail of fluorescently conjugated antibodies. For rhesus samples, cell suspensions were stained with antibodies targeting CD102 (CBR-IC2/2, Biolegend), CD20 (2H7, Biolegend), CD3 (SP34-2, BD Biosciences), CD64 (10.1, Biolegend), CD11b (ICRF44, Biolegend), CD45 (D058-1283, BD), and CD31 (WM59, BD Biosciences). For human samples, cells suspensions were stained with antibodies targeting CD102 (CBR-IC2/2, Biolegend), CD20 (2H7, Biolegend), CD45 (HI30, Biolegend), CD31 (ICRF44, Biolegend), and CD138 (DL101, Biolegend). Following staining, samples were washed 2× with DPBS before being resuspended in cRPMI. CD45 + cells were further enriched from rhesus samples by magnetic enrichment using anti-NHP CD45 microbeads (Miltenyi Biotech). Cell sorting was performed on a Sony SH800 instrument (Sony) using 100um microfluidic sorting chips. Cells were sorted into low-volume fetal bovine serum to maintain higher cell concentrations before being used in downstream applications.

### Rhesus macaque—ELISPOT

Briefly, ELISPOT plates (MSIPSW10, Millipore Sigma) were activated with 70% EtOH before being washed 4x with sterile dH_2_O. Plates were coated overnight with purified anti-human IgG (MT91/145, Mabtech), anti-monkey IgA (polyclonal, Rockland), or anti-human IgA (MT57, Mabtech). Cells to be assayed were plated in duplicate and serially diluted down the ELISPOT plate before overnight incubation at 37 °C in a 5% CO_2_ incubator. The following day, cells were lysed by incubation with dH_2_O before being washed 4× with PBS-T. Secreted IgG or IgA were detected using biotinylated anti-human IgG (MT78/145, Mabtech) or biotinylated anti-human IgA (MT57, Mabtech) followed by incubation with streptavidin-ALP (Mabtech). ELISPOT plates were developed with NBT/BCIP (ThermoFisher Scientific) before being quenched with dH_2_O. All washes between incubation steps were performed using PBS-T. Plates were read and quantified on an Immunospot S6 (Cellular Technology Limited (CTL)) using CTL Immunocapture (v7.0.16.1, CTL) and CTL Biospot (v7.0.3.4, CTL). Manual inspection of automated counting results was performed as quality control during analysis.

### Human—ELISPOT

Human total-IgG and total-IgA ELISPOTs were performed as described above for rhesus macaque ELISPOTs with the following exception. Plates were coated overnight with purified anti-human IgG (MT91/145, Mabtech) or anti-human IgA (MT57, Mabtech). All other detection reagents, wash steps, and data acquisition and analysis were performed as for rhesus macaque samples.

### Single-cell multi-omics—sample preparation

Mononuclear single-cell suspensions were isolated from whole blood, lymph nodes, and bone marrow from 5 rhesus macaques as described above. All samples used were fresh and never cryo-preserved. Accordingly, processing of samples from each rhesus macaque were performed on separate days.

### Single-cell multi-omics—CITEseq antibody panel preparation

A panel of 124 oligo-conjugated antibodies containing 115 antibodies targeting surface-expressed proteins of interest and 9 isotype controls was used in these studies. All antibodies, except 1, were in the TotalSeqA format and available commercially (Biolegend). CD38-bio (clone OKT10, Caprico Biotechnologies) and oligo-conjugated streptavidin-PE (SA-PE) (Biolegend) were used to detect CD38 in this panel. Details on antibodies used and clone information are contained in Supplementary Data 2. All antibodies used were determined to be cross-reactive between human and rhesus using the LEGENDScreen assay (Biolegend).

### Single-cell multi-omics—CITEseq staining

CITEseq staining was performed per manufacturer’s recommendations (Biolegend) with the following exceptions. Briefly, 1 × 10^6^ cells from each of the PBMC, lymph node, and bone marrow single-cell suspensions were placed in 5 ml FACS tubes and washed 1× with Cell Staining Buffer (CSB) (Biolegend). Cells were resuspended in 100ul of CSB, 5ul of Fc block (Human TruStain FcX, Biolegend) was added, and samples were incubated for 15 min at room temperature. During Fc block, the previously prepared CITEseq master mix was centrifuged at 14,000 × *g* at 4 °C for 10 min. Following Fc block, samples were washed with 2 ml of CSB, pelleted by centrifugation at 400 × *g* for 5 min at room temperature, prior to resuspension in 125 ul of CITEseq antibody master mix, and incubation for 30 min at room temperature. Following cell staining, samples were washed 2× with CSB prior to resuspension in 100ul CSB, and incubation with 1 ul of oligo-conjugated SA-PE for 30 min at room temperature in the dark. Cells were washed 2× with 2 ml of CSB prior to final cell counting and loading into the 10x Chromium Instrument (10x Genomics).

### Single-cell multi-omics—assay and sequencing

Stained and counted cell suspensions were loaded into a 10x Chromium instrument (10x Genomics). 10,000 cells from each tissue for each animal were loaded separately in replicate lanes of a Chromium Next GEM Chip G for a combined target of 20,000 cells per tissue per animal. Single-cell multi-omics libraries were prepared per manufacturer’s recommendations (10x Genomics) using the Chromium Next Gem Single Cell 3’ Reagent Kits v3.1. Antibody-derived tag (ADT) libraries were amplified and prepared using the TotalSeqA Reagent Kit v3 3.1 protocol per manufacturer’s recommendations (Biolegend). Prepared libraries were pooled at an 80:20 ratio of RNA:ADT libraries before being sequenced on an Illumina NovaSeq (Genewiz). A total of 100K reads per cell were targeted for the RNA libraries and 25K reads per cell were targeted for the ADT libraries.

### Single-cell multi-omics—alignment and sample quality control

Raw sequencing data were aligned to the rhesus macaque genome (Mmul8, Ensembl v97) and counted using 10x Genomics Cell Ranger 3.1.1 (10x Genomics). Outputs from Cell Ranger were loaded into R (4.0.2) and processed using the Seurat R package (v 4.0.4)^68^. To correct for ambient mRNA contamination, SoupX (v 1.5.2) was used to generate correct RNA count matrices for downstream analysis^69^. Genes detected in fewer than 3 cells were removed from RNA count matrices prior to Seurat object generation and quality control. Low quality cells with less than 250 detected genes (BM samples) or less than 500 detected genes (LN and PBMC) were filtered along with cells containing greater than 15% of reads derived from mitochondrial reads. Outlier cells with total RNA library counts or ADT library 3 median absolute deviations away from the median (both above and below) were also removed. To remove cells that bound a high amount of isotype control antibody the number of counts per cell derived from isotype control antibodies was calculated and cells 3 median absolute deviations away from the median (both above and below) were removed. Doublet prediction was performed with Scrublet (v 0.2.3) using automated thresholding^70^. For samples with a predicted doublet threshold >0.30, the threshold was manually set to 0.25. All predicted doublets were removed before downstream analysis. DSB (v 0.2.0) was used to normalize ADT count matrices for individual libraries prior to merging count matrices from all samples^71^. Merged data and data from each tissue were processed separately as described below.

### Single-cell multi-omics—merged data processing

Cell cycle scores were calculated using previously described cell cycle genes and a score for the “difference” in cell cycle status was calculated according to the Seurat documentation. Sctransform (v 0.3.2) was used to normalize RNA counts with variation due to % of mitochondrial reads and cell cycle “difference” regressed out^72^. Dimensionality reduction of RNA data layer was performed using PCA followed by batch correction using Harmony (v 1.0) using individual animals, individual samples, and tissue of origin as covariates^73^. Dimensionality reduction of DSB-normalized ADT data layer was performed using PCA followed by batch correction using Harmony (v 1.0) using individual animals, individual samples, and tissue of origin as covariates^73^. Harmony-corrected RNA and ADT PCA were used to construct the Weighted Nearest Neighbor graph using the first 27 RNA harmony dimensions and the first 26 ADT harmony dimensions^68^. Data were clustered at resolution of 1.2 using the smart local moving algorithm prior to dimensionality reduction and visualization using UMAP.

### Single-cell multi-omics—bone marrow data processing

Cell cycle scores were calculated using previously described cell cycle genes and a score for the “difference” in cell cycle status was calculated according to the Seurat documentation. Sctransform (v 0.3.2) was used to normalize RNA counts with variation due to % of mitochondrial reads and cell cycle “difference” regressed out^72^. Dimensionality reduction of RNA data layer was performed using PCA followed by batch correction using Harmony (v 1.0) using individual animals, individual samples, and tissue of origin as covariates^73^. Dimensionality reduction of DSB-normalized ADT data layer was performed using PCA followed by batch correction using Harmony (v 1.0) using individual animals and individual samples as covariates^73^. Harmony-corrected RNA and ADT PCA were used to construct the Weighted Nearest Neighbor graph using the first 25 RNA harmony dimensions and the first 22 ADT harmony dimensions^68^. Data were clustered at resolution of 1.0 using the smart local moving algorithm prior to dimensionality reduction and visualization using UMAP.

### Single-cell multi-omics—lymph node data processing

Cell cycle scores were calculated using previously described cell cycle genes and a score for the “difference” in cell cycle status was calculated according to the Seurat documentation. Sctransform (v 0.3.2) was used to normalize RNA counts with variation due to % of mitochondrial reads and cell cycle “difference” regressed out^72^. Dimensionality reduction of RNA data layer was performed using PCA followed by batch correction using Harmony (v 1.0) using individual animals, individual samples, and tissue of origin as covariates^73^. Dimensionality reduction of DSB-normalized ADT data layer was performed using PCA followed by batch correction using Harmony (v 1.0) using individual animals and individual samples as covariates^73^. Harmony-corrected RNA and ADT PCA were used to construct the Weighted Nearest Neighbor graph using the first 26 RNA harmony dimensions and the first 18 ADT harmony dimensions^68^. Data were clustered at resolution of 0.8 using the smart local moving algorithm prior to dimensionality reduction and visualization using UMAP.

### Single-cell multi-omics—PBMC data processing

Cell cycle scores were calculated using previously described cell cycle genes and a score for the “difference” in cell cycle status was calculated according to the Seurat documentation. Sctransform (v 0.3.2) was used to normalize RNA counts with variation due to % of mitochondrial reads and cell cycle “difference” regressed out^72^. Dimensionality reduction of RNA data layer was performed using PCA followed by batch correction using Harmony (v 1.0) using individual animals, individual samples, and tissue of origin as covariates^73^. Dimensionality reduction of DSB-normalized ADT data layer was performed using PCA followed by batch correction using Harmony (v 1.0) using individual animals and individual samples as covariates^73^. Harmony-corrected RNA and ADT PCA were used to construct the Weighted Nearest Neighbor graph using the first 22 RNA harmony dimensions and the first 21 ADT harmony dimensions^68^. Data were clustered at resolution of 0.8 using the smart local moving algorithm prior to dimensionality reduction and visualization using UMAP.

### Data annotation

Clusters were manually annotated into cell lineages using canonical marker genes for immune cell types and hematopoiesis. Differential expression analysis was performed using the wilcoxauc function from the presto R package (v 1.0.0) comparing each cluster versus all other clusters (github.com/immunogenomics/presto). Genes or proteins were considered differentially expressed if they were detected in more than 10% of cells in the query and comparison clusters, were differentially expressed at a 0.25 logFC cut-off, and had a padj value <0.01. Fine cluster annotations were assigned based on differentially expressed markers both by magnitude of log fold-change and AUC score. RNA and ADT features were used to assign fine cell annotations. Clusters containing multiple marker genes from major cell lineages (i.e., *CD3E* from T cells and *MS4A1* from B cells) were removed. Additionally, small clusters visually contained within a larger cell cluster were merged with the larger cell cluster. Differential expression analysis was repeated after doublet clusters were removed and small clusters were merged for the final dataset. This analysis was repeated for the merged dataset and for each individual tissue dataset.

### Statistics and reproducibility

All statistical analyses were performed in R (v 4.0.2) or Python (v3.7) using the packages specified within the materials and methods sections or within GraphPad Prism (v9.0.0). All source data used to produce the graphs and charts within this manuscript are described in the methods, the custom analysis code (available upon reasonable request), or are provided in Supplementary Data 7. All experiments in this manuscript were repeated independently a minimum of two times. For antibody cross-reactivity screen, LEGENDScreen assay was performed for two independent biological animals. For single-cell multi-omic profiling experiments, samples were collected from a total of five individual animals across five independent experiments and replicates were technical replicates. For all NHP flow cytometry and cell sorting experiments, samples from four-five individual NHP were used across four-five independent experiments. For mouse plasma cell CD102 screen, samples from five individual mice were used. For human bone marrow cell sorting experiments, samples from a total of 4 individual biological donors were obtained in two independent experiments. All ELISPOT assays were conducted using technical replicates for each animal/donor sample. Precise n’s used in each experiment and details about statistics calculated are noted in the figure legends.

### Reporting summary

Further information on research design is available in the Nature Portfolio Reporting Summary linked to this article.

## Supplementary information

Supplementary Information

Description of Additional Supplementary Files

Supplementary Data 1

Supplementary Data 2

Supplementary Data 3

Supplementary Data 4

Supplementary Data 5

Supplementary Data 6

Supplementary Data 7

Reporting Summary

## Data Availability

All raw and processed genomics data generated in this project have been deposited in the NCBI’s Gene Expression Omnibus (GEO)^74,75^ and are accessible through GEO Series accession number GSE216456. Source data for all graphs presenting a mean and error are available in Supplementary Data 7. All other data not included in the manuscript or supplementary materials are available upon reasonable request to the corresponding author.

## Code availability

Custom code for data processing and analysis are available upon reasonable request to the study authors R.P.S. (ryan.staupe@merck.com) and N.L.S. (nicole.sullivan@merck.com).
