# Supplementary material for: Single cell multi-omic reference atlases of non-human primate immune tissues reveals CD102 as a biomarker for long-lived plasma cells
Source: Commun Biol. 2022 Dec 21;5:1399. doi: 10.1038/s42003-022-04216-9 (PMC9770566; doi:10.1038/s42003-022-04216-9)
Supplement: Supplementary file 1 — Supplementary Information [file 42003_2022_4216_MOESM1_ESM.pdf]

Supplementary Information: Supplementary Figures 1-7

# Single cell multi-omic reference atlases of non-human primate immune tissues reveals CD102 as a biomarker for long-lived plasma cells.

Ryan P. Staupe[1], Kenneth E. Lodge[2], Nithya Thambi[1], David Toole[3,4], Alex M. Tamburino[5], Dan Chang[6,7], Bonnie J. Howell[1,8], Daria J. Hazuda[1,9], Kalpit A. Vora[1], Nicole L. Sullivan[1]*

[1]Infectious Diseases and Vaccines, MRL, Merck & Co., Inc., West Point, PA, USA

[2]LAR, Integrative Vet Medicine, MRL, Merck & Co., Inc., West Point, PA, USA

[3]Atlas Data Systems, Berkeley Heights, NJ, USA

[4]Large Data Delivery Services, MRL, Merck & Co., Inc., West Point, PA, USA

[5]Genome & Biomarker Sciences, MRL, Merck & Co., Inc., West Point, PA, USA

[6]Genome & Biomarker Sciences, MRL, Merck & Co., Inc., Cambridge, MA, USA

[7]Current address: AbbVie Inc, Cambridge, MA, USA

[8]Current affiliation: Quantitative Biosciences, MRL, Merck & Co., Inc., West Point, PA, USA

[9]Current affiliation: Vaccine & Medical Affairs, Merck & Co., Inc., West Point, PA, USA

*Corresponding author. Email: nicole.sullivan@merck.com

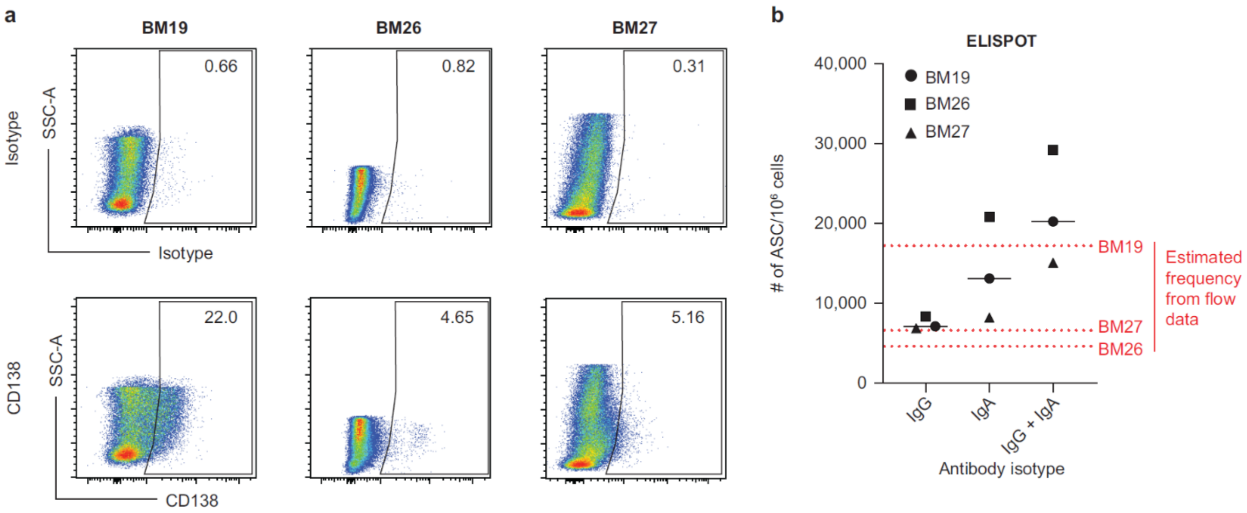
a
BM19
BM26
BM27
Isotype
SSC-A
0.66
0.82
0.31
Isotype
CD138
SSC-A
22.0
4.65
5.16
CD138
b
ELISPOT
40,000
30,000
20,000
10,000
0
BM19
BM26
BM27
# of ASC/10⁶ cells
BM19
BM27
BM26
Estimated frequency from flow data
IgG
IgA
IgG + IgA
Antibody isotype

**Supplementary Figure 1. CD138 staining in NHP BM.** (**a**) Flow cytometry plots depicting frequency of cells expressing CD138 in NHP BM. Cells were previously gated as CD20-CD3-CD45+ live lymphocytes. Binding of mIgG1 isotype control and anti-CD138 (clone DL101) are shown for three animals. (**b**) Quantification of BM IgG and IgA antibody-secreting cells (ASC) within NHP bone marrow. Data shown are from the three animals in panel A. IgG + IgA is the sum of each isotype alone. Dashed lines in red depict estimated quantification of LLPC frequency as measured by flow cytometry for each animal.

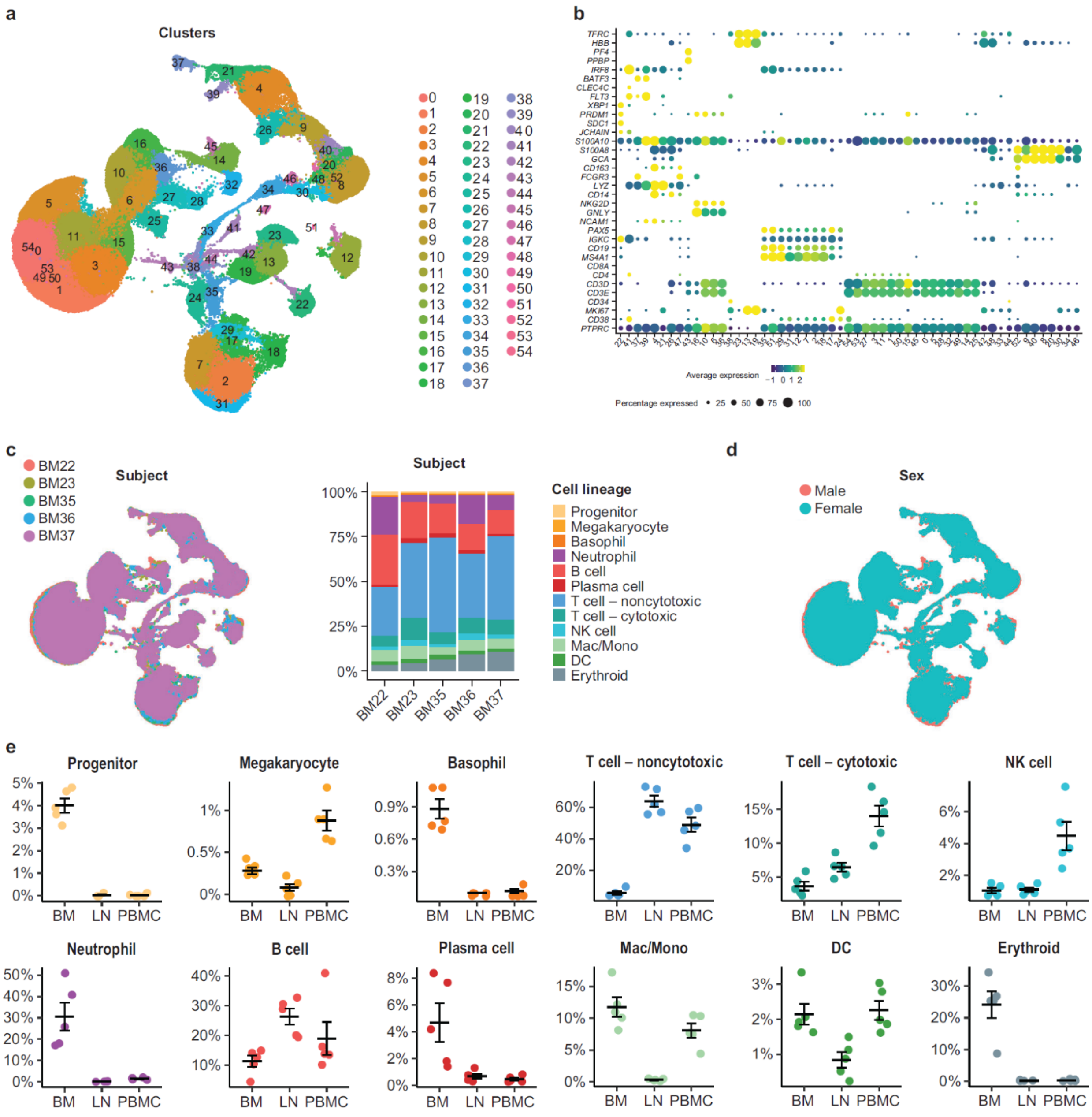
a
Clusters
b
TFRC
HBB
PF4
PPBP
IRF8
BATF3
CLEC4C
FLT3
XBP1
PRDM1
SDC1
JCHAIN
S100A10
S100A8
GCA
CD163
FCGR3
LYZ
CD14
NKG2D
GNLY
NCAM1
PAX5
IGKC
CD19
MS4A1
CD8A
CD4
CD3D
CD3E
CD34
MKI67
CD38
PTPRC
Average expression
Percentage expressed
c
BM22
BM23
BM35
BM36
BM37
Subject
Subject
Cell lineage
Progenitor
Megakaryocyte
Basophil
Neutrophil
B cell
Plasma cell
T cell – noncytotoxic
T cell – cytotoxic
NK cell
Mac/Mono
DC
Erythroid
d
Male
Female
Sex
e
Progenitor
Megakaryocyte
Basophil
T cell – noncytotoxic
T cell – cytotoxic
NK cell
Neutrophil
B cell
Plasma cell
Mac/Mono
DC
Erythroid
BM
LN
PBMC

**Supplementary Figure 2: Merged single cell atlas data annotation** (**a**) Results from unbiased clustering of cells from rhesus PBMC, LN, and BM visualized as a UMAP plot. 55 unique clusters of cells were identified. (**b**) Expression of canonical immune cell type marker genes for each of the cell clusters identified during unbiased clustering. (**c**) Left panel: UMAP visualization of cells from rhesus PBMC, LN, and BM colored by subject. Right panel: distribution of major cell lineages across each subject. (**d**) UMAP visualization of cells from rhesus PBMC, LN, and BM colored by sex. (**e**) Distribution of major cell lineages across each tissue and biological replicate. Percentages represent the proportion of the indicated cell type for each subject within the three tissues compartments assessed as a fraction of the total number of cells for each subject within a tissue. Error bars represent standard error of the mean (SEM). n=5 individual biological animals.

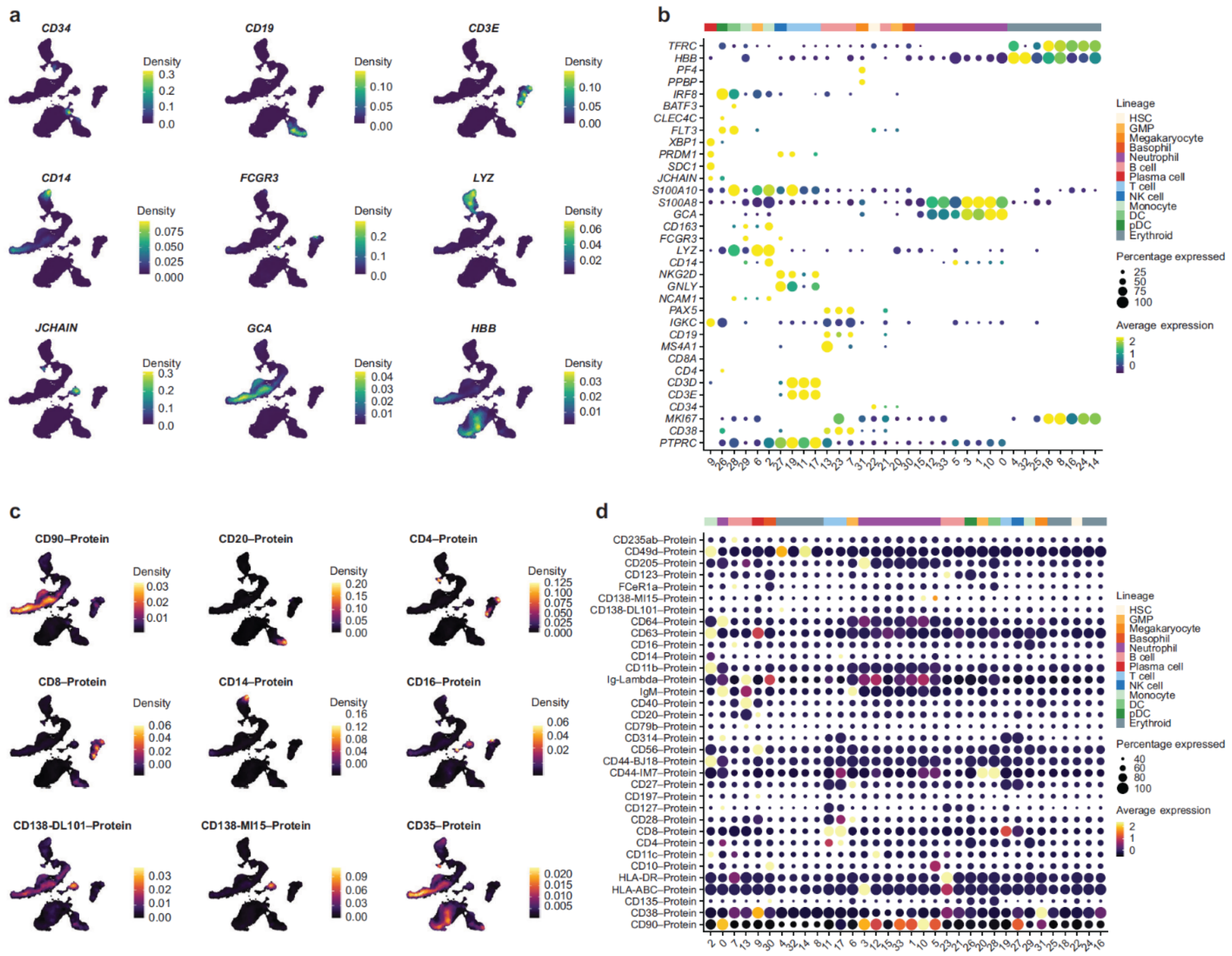

a
CD34
CD19
CD3E
CD14
FCGR3
LYZ
JCHAIN
GCA
HBB
Density
b
TFRC
HBB
PF4
PPBP
IRF8
BATF3
CLEC4C
FLT3
XBP1
PRDM1
SDC1
JCHAIN
S100A10
S100A8
GCA
CD163
FCGR3
LYZ
CD14
NKG2D
GNLY
NCAM1
PAX5
IGKC
CD19
MS4A1
CD8A
CD4
CD3D
CD3E
CD34
MKI67
CD38
PTPRC
Lineage
HSC
GMP
Megakaryocyte
Basophil
Neutrophil
B cell
Plasma cell
T cell
NK cell
Monocyte
DC
pDC
Erythroid
Percentage expressed
25
50
75
100
Average expression
2
1
0
c
CD90–Protein
CD20–Protein
CD4–Protein
CD8–Protein
CD14–Protein
CD16–Protein
CD138-DL101–Protein
CD138-MI15–Protein
CD35–Protein
Density
d
CD235ab–Protein
CD49d–Protein
CD205–Protein
CD123–Protein
FCeR1a–Protein
CD138-MI15–Protein
CD138-DL101–Protein
CD64–Protein
CD63–Protein
CD16–Protein
CD14–Protein
CD11b–Protein
Ig-Lambda–Protein
IgM–Protein
CD40–Protein
CD20–Protein
CD79b–Protein
CD314–Protein
CD56–Protein
CD44-BJ18–Protein
CD44-IM7–Protein
CD27–Protein
CD197–Protein
CD127–Protein
CD28–Protein
CD8–Protein
CD4–Protein
CD11c–Protein
CD10–Protein
HLA-DR–Protein
HLA-ABC–Protein
CD135–Protein
CD38–Protein
CD90–Protein
Lineage
HSC
GMP
Megakaryocyte
Basophil
Neutrophil
B cell
Plasma cell
T cell
NK cell
Monocyte
DC
pDC
Erythroid
Percentage expressed
40
60
80
100
Average expression
2
1
0

**Supplementary Figure 3: Annotation of NHP BM single cell reference atlas.** (**a**) UMAP visualization of the expression of major immune cell lineage genes at the RNA level. Expression is visualized as the kernel density estimate for each RNA. (**b**) Expression of canonical immune cell type marker genes for each of the cell clusters identified during unbiased clustering. (**c**) UMAP visualization of the major immune cell lineage genes at the protein level. Expression is visualized as the kernel density estimate for each protein. (**d**) Expression of canonical immune cell type surface protein marker for each of the cell clusters identified during unbiased clustering.

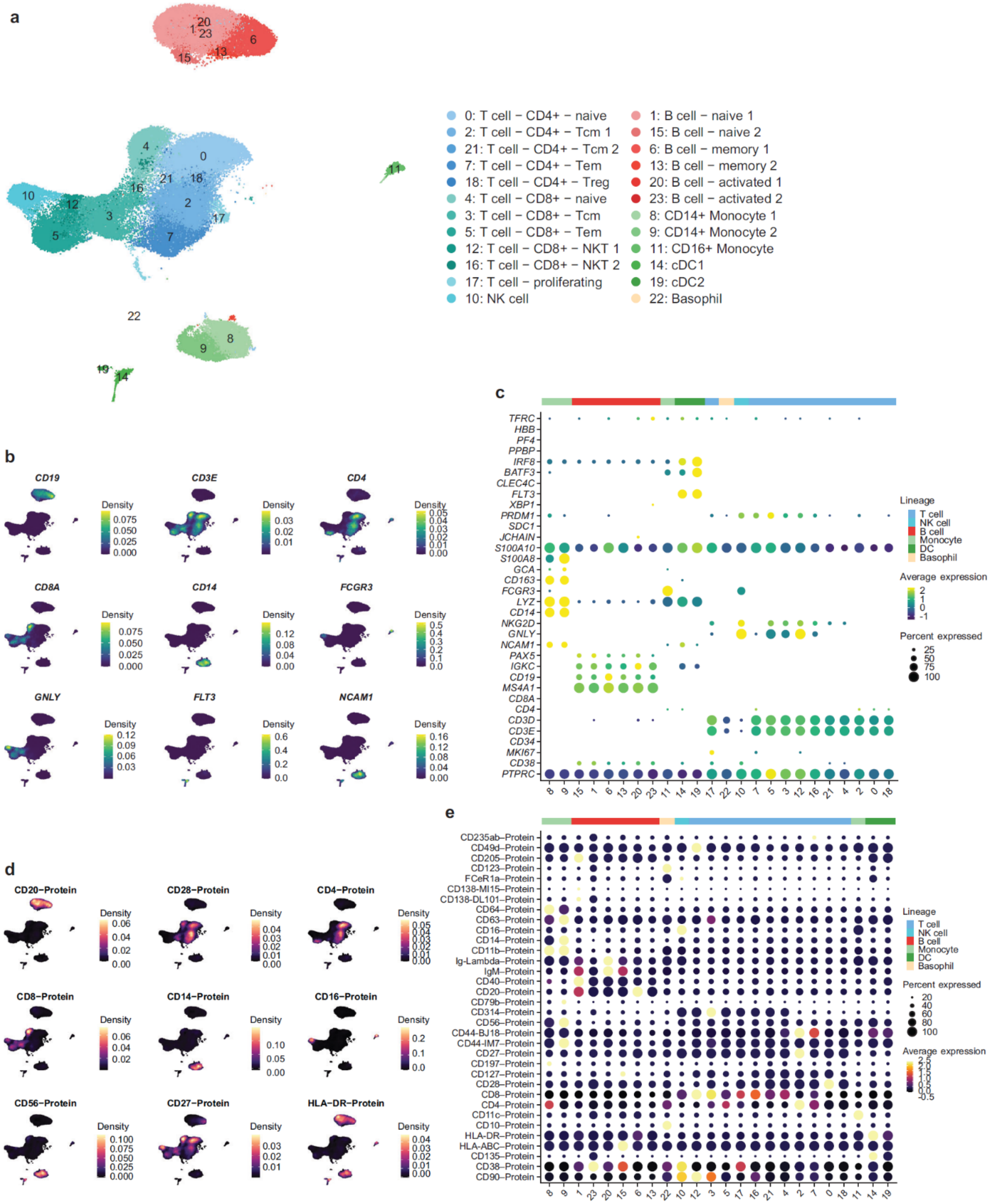
a
0: T cell − CD4+ − naive
2: T cell − CD4+ − Tcm 1
21: T cell − CD4+ − Tcm 2
7: T cell − CD4+ − Tem
18: T cell − CD4+ − Treg
4: T cell − CD8+ − naive
3: T cell − CD8+ − Tcm
5: T cell − CD8+ − Tem
12: T cell − CD8+ − NKT 1
16: T cell − CD8+ − NKT 2
17: T cell − proliferating
10: NK cell
1: B cell − naive 1
15: B cell − naive 2
6: B cell − memory 1
13: B cell − memory 2
20: B cell − activated 1
23: B cell − activated 2
8: CD14+ Monocyte 1
9: CD14+ Monocyte 2
11: CD16+ Monocyte
14: cDC1
19: cDC2
22: Basophil
b
CD19
CD3E
CD4
CD8A
CD14
FCGR3
GNLY
FLT3
NCAM1
Density
c
TFRC
HBB
PF4
PPBP
IRF8
BATF3
CLEC4C
FLT3
XBP1
PRDM1
SDC1
JCHAIN
S100A10
S100A8
GCA
CD163
FCGR3
LYZ
CD14
NKG2D
GNLY
NCAM1
PAX5
IGKC
CD19
MS4A1
CD8A
CD4
CD3D
CD3E
CD34
MKI67
CD38
PTPRC
Lineage
T cell
NK cell
B cell
Monocyte
DC
Basophil
Average expression
Percent expressed
d
CD20−Protein
CD28−Protein
CD4−Protein
CD8−Protein
CD14−Protein
CD16−Protein
CD56−Protein
CD27−Protein
HLA−DR−Protein
Density
e
CD235ab−Protein
CD49d−Protein
CD205−Protein
CD123−Protein
FCeR1a−Protein
CD138−MI15−Protein
CD138−DL101−Protein
CD64−Protein
CD63−Protein
CD16−Protein
CD14−Protein
CD11b−Protein
Ig−Lambda−Protein
IgM−Protein
CD40−Protein
CD20−Protein
CD79b−Protein
CD314−Protein
CD56−Protein
CD44−BJ18−Protein
CD44−IM7−Protein
CD27−Protein
CD197−Protein
CD127−Protein
CD28−Protein
CD8−Protein
CD4−Protein
CD11c−Protein
CD10−Protein
HLA−DR−Protein
HLA−ABC−Protein
CD135−Protein
CD38−Protein
CD90−Protein
Lineage
T cell
NK cell
B cell
Monocyte
DC
Basophil
Percent expressed
Average expression

**Supplementary Figure 4: Single cell reference atlas of NHP PBMC.** (**a**) UMAP visualization of cells from NHP PBMC depicting results of unbiased clustering and cell type annotation. (**b**) UMAP visualization of the expression of major immune cell lineage genes at the RNA level. (**c**) Expression of canonical immune cell type marker genes for each of the cell clusters identified during unbiased clustering. (**d**) UMAP visualization of the major immune cell lineage genes at the protein level. Expression is visualized as the kernel density estimate for each protein. (**e**) Expression of canonical immune cell type surface protein marker for each of the cell clusters identified during unbiased clustering.

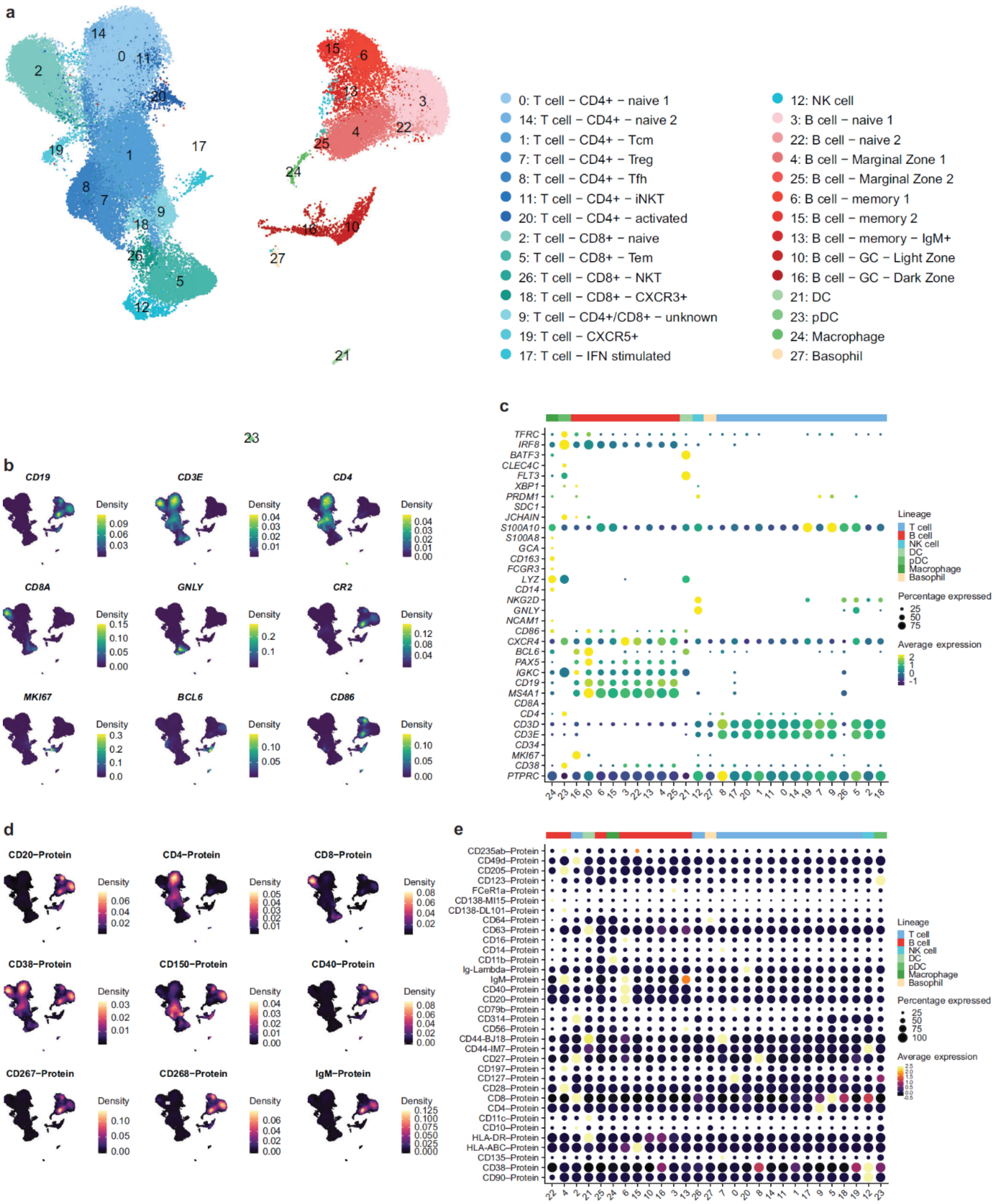

a
0: T cell – CD4+ – naive 1
14: T cell – CD4+ – naive 2
1: T cell – CD4+ – Tcm
7: T cell – CD4+ – Treg
8: T cell – CD4+ – Tfh
11: T cell – CD4+ – iNKT
20: T cell – CD4+ – activated
2: T cell – CD8+ – naive
5: T cell – CD8+ – Tem
26: T cell – CD8+ – NKT
18: T cell – CD8+ – CXCR3+
9: T cell – CD4+/CD8+ – unknown
19: T cell – CXCR5+
17: T cell – IFN stimulated
12: NK cell
3: B cell – naive 1
22: B cell – naive 2
4: B cell – Marginal Zone 1
25: B cell – Marginal Zone 2
6: B cell – memory 1
15: B cell – memory 2
13: B cell – memory – IgM+
10: B cell – GC – Light Zone
16: B cell – GC – Dark Zone
21: DC
23: pDC
24: Macrophage
27: Basophil
b
CD19
CD3E
CD4
CD8A
GNLY
CR2
MKI67
BCL6
CD86
Density
c
Lineage
T cell
B cell
NK cell
DC
pDC
Macrophage
Basophil
Percentage expressed
Average expression
d
CD20–Protein
CD4–Protein
CD8–Protein
CD38–Protein
CD150–Protein
CD40–Protein
CD267–Protein
CD268–Protein
IgM–Protein
e

**Supplementary Figure 5: Single cell reference atlas of NHP LN.** (**a**) UMAP visualization of cells from NHP LN depicting results of unbiased clustering and cell type annotation. (**b**) UMAP visualization of the expression of major immune cell lineage genes at the RNA level. (**c**) Expression of canonical immune cell type marker genes for each of the cell clusters identified during unbiased clustering. (**d**) UMAP visualization of the major immune cell lineage genes at the protein level. Expression is visualized as the kernel density estimate for each protein. (**e**) Expression of canonical immune cell type surface protein marker for each of the cell clusters identified during unbiased clustering.

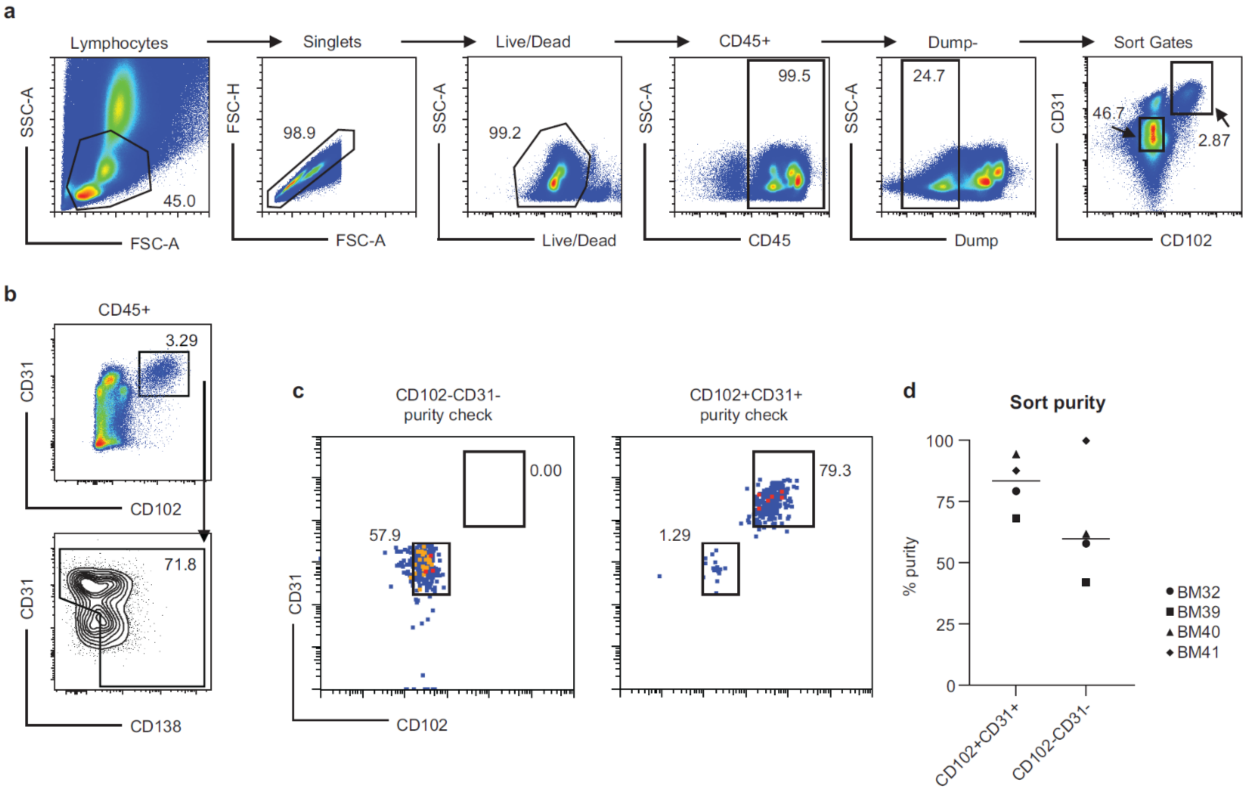
a
Lymphocytes
Singlets
Live/Dead
CD45+
Dump-
Sort Gates
SSC-A
FSC-A
45.0
FSC-H
98.9
99.2
99.5
24.7
CD31
46.7
2.87
CD45
Dump
CD102
b
CD45+
3.29
71.8
CD138
c
CD102-CD31-
purity check
CD102+CD31+
purity check
0.00
57.9
79.3
1.29
d
Sort purity
% purity
100
75
50
25
0
BM32
BM39
BM40
BM41
CD102+CD31+
CD102-CD31-

**Supplementary Figure 6: NHP BM CD102 and CD31 sort quality control.** (**a**) Representative gating strategy to identify and sort CD102+CD31+ and CD102-CD31- cells from NHP BM. Sorted cells were gated as CD45+Dump- live lymphocytes prior to CD102 and CD31 sort gates. Dump channel comprised of antibodies against CD3, CD20, CD11b, and CD64. (**b**) Top panel: Representative plot depicting the frequency of CD102+CD31+ cells in the CD45+ fraction of NHP bone marrow. Bottom panel: Frequency of CD138+Intracellular IgG+ cells within the CD102+CD31+ fraction. (**c**) Representative purities of sorted CD102+CD31+ and CD102-CD31- populations from one NHP BM sample. Numbers depicted are the frequency of cells within the respective gate. (**d**) Sort purities for all sorted NHP BM samples for the CD102+CD31+ and CD102-CD31- populations. Individual animals are depicted by unique symbols. n=4 individual biological animals.

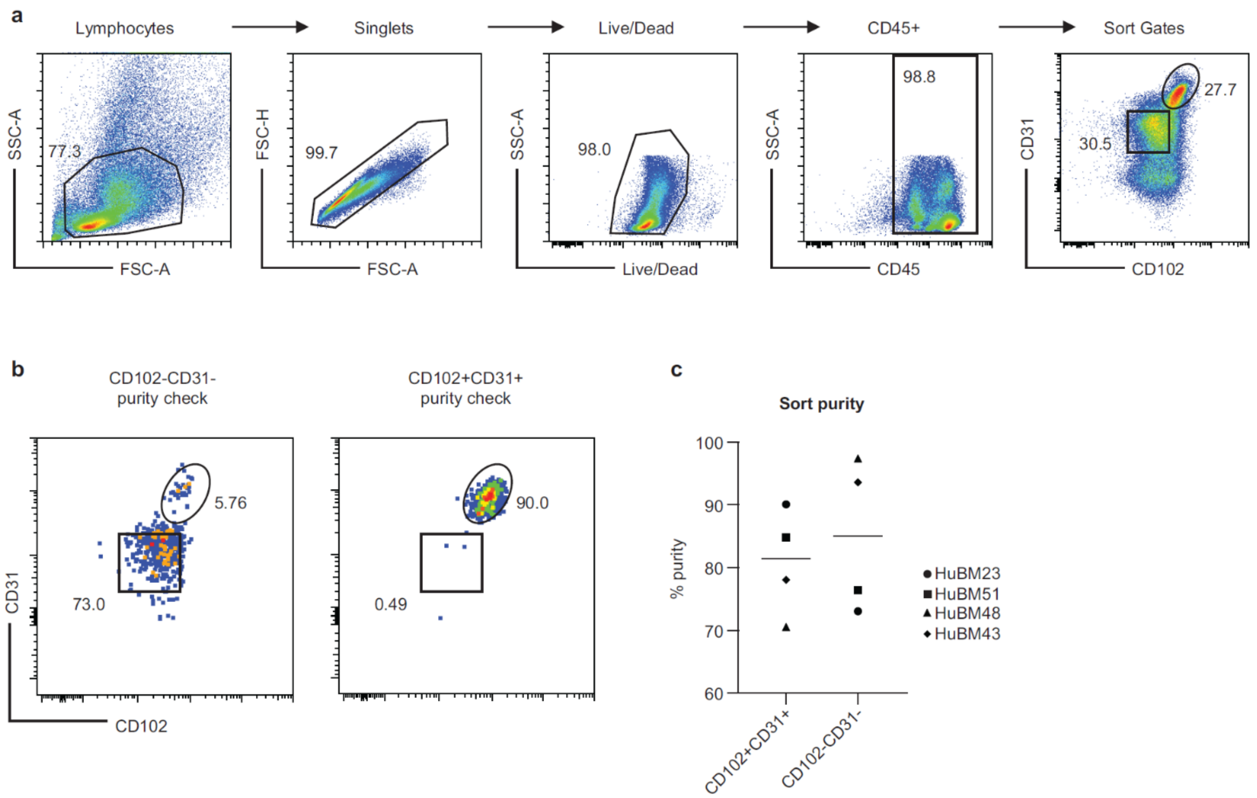
a
Lymphocytes
Singlets
Live/Dead
CD45+
Sort Gates
SSC-A
FSC-A
77.3
FSC-H
99.7
98.0
SSC-A
Live/Dead
98.8
CD45
CD31
CD102
30.5
27.7
b
CD102-CD31-
purity check
CD102+CD31+
purity check
5.76
73.0
90.0
0.49
c
Sort purity
% purity
100
90
80
70
60
CD102+CD31+
CD102-CD31-
HuBM23
HuBM51
HuBM48
HuBM43

**Supplementary Figure 7: Human BM CD102 and CD31 sort quality control.** (**a**) Representative gating strategy to identify and sort CD102+CD31+ and CD102-CD31- cells from fresh human BM aspirates. Sorted cells were gated as CD45+ live lymphocytes prior to CD102 and CD31 sort gates. (**b**) Representative purities of sorted CD102+CD31+ and CD102-CD31- populations from one human BM sample. Numbers depicted are the frequency of cells within the respective gate. (**c**) Sort purities for all sorted human BM samples for the CD102+CD31+ and CD102-CD31- populations. Individual subjects are depicted by unique symbols. n=4 individual human subjects.
