# Supplementary material for: Single cell multi-omic reference atlases of non-human primate immune tissues reveals CD102 as a biomarker for long-lived plasma cells
Source: Commun Biol. 2022 Dec 21;5:1399. doi: 10.1038/s42003-022-04216-9 (PMC9770566; doi:10.1038/s42003-022-04216-9)
Supplement: Supplementary file 2 — Description of Additional Supplementary Files [file 42003_2022_4216_MOESM2_ESM.pdf]

Description of Additional Supplementary Files

File name: Supplementary Data 1
Description: Results from human-rhesus antibody cross-reactivity screen

File name: Supplementary Data 2
Description: Antibodies used for single-cell surface protein profiling and flow cytometry experiments

File name: Supplementary Data 3
Description: Cluster marker genes and proteins for merged reference atlas

File name: Supplementary Data 4
Description: Cluster marker genes and proteins for BM reference atlas

File name: Supplementary Data 5
Description: Cluster marker genes and proteins for PBMC reference atlas

File name: Supplementary Data 6
Description: Cluster marker genes and proteins for LN reference atlas

File name: Supplementary Data 7
Description: Source data for charts and graphs within this manuscript
