# Supplementary material for: Single cell multi-omic reference atlases of non-human primate immune tissues reveals CD102 as a biomarker for long-lived plasma cells
Source: Commun Biol. 2022 Dec 21;5:1399. doi: 10.1038/s42003-022-04216-9 (PMC9770566; doi:10.1038/s42003-022-04216-9)
Supplement: Supplementary file 10 — Reporting Summary [file 42003_2022_4216_MOESM10_ESM.pdf]

nature research

Corresponding author(s): Dr. Nicole Sullivan and Dr. Ryan Staupe

Last updated by author(s): Oct 25, 2022

Reporting Summary

Nature Research wishes to improve the reproducibility of the work that we publish. This form provides structure for consistency and transparency in reporting. For further information on Nature Research policies, see our Editorial Policies and the Editorial Policy Checklist.

Statistics

For all statistical analyses, confirm that the following items are present in the figure legend, table legend, main text, or Methods section.

n/a	Confirmed	
☐	☒	The exact sample size (n) for each experimental group/condition, given as a discrete number and unit of measurement
☐	☒	A statement on whether measurements were taken from distinct samples or whether the same sample was measured repeatedly
☐	☒	The statistical test(s) used AND whether they are one- or two-sided<br>*Only common tests should be described solely by name; describe more complex techniques in the Methods section.*
☐	☒	A description of all covariates tested
☐	☒	A description of any assumptions or corrections, such as tests of normality and adjustment for multiple comparisons
☐	☒	A full description of the statistical parameters including central tendency (e.g. means) or other basic estimates (e.g. regression coefficient) AND variation (e.g. standard deviation) or associated estimates of uncertainty (e.g. confidence intervals)
☐	☒	For null hypothesis testing, the test statistic (e.g. F, t, r) with confidence intervals, effect sizes, degrees of freedom and P value noted<br>*Give P values as exact values whenever suitable.*
☒	☐	For Bayesian analysis, information on the choice of priors and Markov chain Monte Carlo settings
☒	☐	For hierarchical and complex designs, identification of the appropriate level for tests and full reporting of outcomes
☒	☐	Estimates of effect sizes (e.g. Cohen's d, Pearson's r), indicating how they were calculated

Our web collection on statistics for biologists contains articles on many of the points above.

Software and code

Policy information about availability of computer code

Data collection	FACSDiva (BD Biosciences), Sony Cell Sorter Software (v2.1.5 Sony), CTL ImmunoCapture (v7.0.16.1, Cellular Technology Limited), CTL BioSpot (v7.0.3.4, Cellular Technology Limited)
Data analysis	FlowJo (v 10.6.2, BD Biosciences), GraphPad (v9.0.0), R (v4.0.2), Python (v3.7), 10x Genomics Cell Ranger 3.1.1 (10x Genomics), Seurat R package (v 4.0.4), SoupX (v 1.5.2), Scrublet (v 0.2.3), DSB (v 0.2.0), Sctransform (v 0.3.2), Harmony (v 1.0), presto R package (v 1.0.0)

For manuscripts utilizing custom algorithms or software that are central to the research but not yet described in published literature, software must be made available to editors and reviewers. We strongly encourage code deposition in a community repository (e.g. GitHub). See the Nature Research guidelines for submitting code & software for further information.

Data

Policy information about availability of data

All manuscripts must include a data availability statement. This statement should provide the following information, where applicable:

- Accession codes, unique identifiers, or web links for publicly available datasets
- A list of figures that have associated raw data
- A description of any restrictions on data availability

All raw and processed genomics data generated in this project have been deposited in the NCBI's Gene Expression Omnibus (GEO) 74,75 and are accessible through GEO Series accession number GSE216456 (https://www.ncbi.nlm.nih.gov/geo/query/acc.cgi?acc=GSE216456). Source data for all graphs presenting a mean and error are available in Supplementary Data 7. All other data not included in the manuscript or supplementary materials is available upon reasonable request to the corresponding author.

Field-specific reporting

Please select the one below that is the best fit for your research. If you are not sure, read the appropriate sections before making your selection.

☒ Life sciences ☐ Behavioural & social sciences ☐ Ecological, evolutionary & environmental sciences

For a reference copy of the document with all sections, see nature.com/documents/nr-reporting-summary-flat.pdf

Life sciences study design

All studies must disclose on these points even when the disclosure is negative.

Sample size	No sample size calculation was performed for these studies. A total of 5 NHP were chosen of varying age and sex which we believe would be representative of most NHP tissue samples obtained from healthy rhesus macaque.
Data exclusions	No data were excluded from these studies
Replication	Human-rhesus cross reactivity data are show and are representative of n=2 experiments performed for a total of 2 NHP profiled.<br>For single cell analysis, 5 individual NHP were profiled and biological replicates were run for each tissue within each subject. Biological replicate samples were treated as unique samples throughout processing and analysis. Major batch effects were not observed between replicates from the same NHP and proportion of major cell types found within a tissue between replicates were consistent within individual NHPs.<br><br>Flow cytometry and flow sorting experiments were conducted with 4-5 replicate mice, human bone marrow samples, or NHP bone marrow samples. Attempts at replication were successful for these experiments.
Randomization	All samples were obtained without prior inclusion/exclusion criteria and therefore are randomized for all experiments conducted.
Blinding	Blinding was not needed for these studies because all samples profiled came from otherwise healthy animals or subjects and no specific comparison was made regarding subject level metadata, and all results presented in this work do not make claims about any specific healthy or disease outcome.

Reporting for specific materials, systems and methods

We require information from authors about some types of materials, experimental systems and methods used in many studies. Here, indicate whether each material, system or method listed is relevant to your study. If you are not sure if a list item applies to your research, read the appropriate section before selecting a response.

Materials & experimental systems

n/a	Involved in the study	
☐	☒	Antibodies
☒	☐	Eukaryotic cell lines
☒	☐	Palaeontology and archaeology
☐	☒	Animals and other organisms
☐	☒	Human research participants
☒	☐	Clinical data
☒	☐	Dual use research of concern

Methods

n/a	Involved in the study	
☒	☐	ChIP-seq
☐	☒	Flow cytometry
☒	☐	MRI-based neuroimaging

Antibodies

Antibodies used	Information on antibodies used in these studies are available in Supplementary Table 1 and Supplementary Table 2 and the methods second of this manuscript.
Validation	Reactivity of all antibody reagents used in these studies are based off of manufacturer's reported information on manufacturer's website.

Animals and other organisms

Policy information about studies involving animals; ARRIVE guidelines recommended for reporting animal research

Laboratory animals	Indian-origin rhesus macaques were used for these studies and additional details of NHP used in these studies can be found in the table in Figure 2B. Female 6-8 weeks old C57BL6N (Charles River Laboratories) mice were used for these studies.
Wild animals	This study did not involve wild animals.

Field-collected samples	This study did not involve samples collected from the field.
Ethics oversight	All animal studies were carried out by the Research Laboratories of Merck & Co., Inc., Kenilworth, NJ, USA at our West Point, PA, USA and all experiments involving laboratory animals were approved by the Institutional Animal Care and Use Committee (IACUC) of Merck & Co., Inc., Kenilworth, NJ, USA.

Note that full information on the approval of the study protocol must also be provided in the manuscript.

Human research participants

Policy information about studies involving human research participants

Population characteristics	Fresh whole bone marrow aspirates from healthy donors were obtained from the Stem Cell and Xenograft Core at the Perelman School of Medicine, University of Pennsylvania. No enrollment criteria were used for sample collection other than normal healthy tissues from either sex of any age for a consenting study participant.
Recruitment	Study recruitment was done on a volunteer basis at the Perelman School of Medicine, University of Pennsylvania.
Ethics oversight	The informed consent to collect and use human donor-derived specimens for research was obtained under the protocol "Normal Donor Human Bone Marrow Donation For Research", which is approved by the University of Pennsylvania's Institutional Review Board (IRB protocol #701582).

Note that full information on the approval of the study protocol must also be provided in the manuscript.

Flow Cytometry

Plots

Confirm that:

- [ ] The axis labels state the marker and fluorochrome used (e.g. CD4-FITC).
- [x] The axis scales are clearly visible. Include numbers along axes only for bottom left plot of group (a 'group' is an analysis of identical markers).
- [x] All plots are contour plots with outliers or pseudocolor plots.
- [x] A numerical value for number of cells or percentage (with statistics) is provided.

Methodology

Sample preparation

Rhesus macaque sample processing
PBMC processing
Whole blood was collected into either CPT tubes containing sodium heparin (BD Biosciences) or blood collection tubes containing sodium heparin (BD Biosciences). CPT tubes were spun at 1600 x g for 25 minutes with 3 acceleration and no brake at room temperature to separate cell layers. After centrifugation, ~3ml of plasma was collected prior to collecting the cell layer. PBMC from each CPT tube were pooled prior to being washed with DPBS and pellete
d at 400 x g. For blood collection tubes, blood was collected from each tube and pooled prior to being aliquoted into 50ml conicals and underlaid with a layer of room temperature Histopaque-1077 (Sigma Diagnostics). Samples were centrifuged at 800 x g for 15 minutes with 3 acceleration and no brake at room temperature to separate cell layers. Following centrifugation, plasma was collected prior to harvesting PBMC from each CPT tube were pooled prior to being washed with DPBS and pelleted at 400 x g. All PBMC samples were subjected to RBC lysis in ACK Lysis Buffer (Gibco) and strained through a 100um filter (Corning) prior to final resuspension in cRPMI and cell counting. All samples were processed and stored at room temperature unless otherwise noted.
Lymph node processing
Axial, brachial, mesenteric, and inguinal lymph nodes were collected from rhesus macaques < 2 hours post-necropsy. All lymph nodes collected were pooled together in cRPMI prior to tissue processing. To isolate cells from lymph nodes, collected lymph nodes were trimmed of excess fat, placed in a small amount of cRPMI within a petri dish, mechanically dissociated using dissection scissors to disrupt the lymph node capsule, then mechanically dissociated using the flat end of the plunger from a 30ml syringe (BD Biosciences). Isolated cells were harvested from the petri dish before being strained through a 100um filter and washed 3x with DPBS. Mononuclear cells were isolated by underlaying the isolated cell suspension with a layer of room temperature 96% Ficoll (GE Healthcare) prior to centrifugation at 400 x g for 30 minutes with 3 acceleration and no brake. Following centrifugation, mononuclear cells were harvested and washed with DPBS before RBC lysis was performed in ACK Lysis Buffer (Gibco). Cells were resuspended in cRPMI prior to cell counting. All samples were processed and stored at room temperature unless otherwise noted.
Bone marrow processing
Whole femur bone marrow was collected from rhesus macaques < 2 hours post-necropsy. Whole femur marrow as harvested into sodium citrate containing tubes (BD Biosciences). Cells were isolated from bone marrow samples by placing sample on top of a 100um cell strainer filter (Corning) followed by mechanical dissociation using the flat end of a 5ml syringe (BD Biosciences). Cells were washed with DPBS and then underlaid with room temperature 96% Ficoll (GE Healthcare) prior to centrifugation at 400 x g for 30 minutes with 3 acceleration and no brake. Following centrifugation, mononuclear cells were harvested and washed with DPBS before RBC lysis was performed in ACK Lysis Buffer (Gibco). Cells were resuspended in cRPMI prior to cell counting. All samples were processed and stored at room temperature unless otherwise noted.

Mouse sample processing
Bone marrow processing

Bone marrow was harvested from the femur and tibia by isolating the leg bones, snipping the top and bottom of each bone to expose the bone marrow, and flushing the bone marrow with cRPMI using a 5ml syringe fitted with a G18 needle. Isolated bone marrows were pelleted at 500 x g for 10 minutes prior to resuspension in cRPMI and filtration through a 100um cell strainer set over a 50ml conical. Cells that did not flow through the filter were mechanically dissociated using the flat end of the plunger from a 5ml syringe (BD Biosciences) prior to washing with DPBS. Cells were pelleted by centrifugation at 500 x g for 5 minutes prior to RBC lysis with ACK Lysis Buffer (Gibco). Cells were resuspended in cRPMI prior to cell counting. All samples were processed at room temperature and stored at 4C unless otherwise noted.

Human sample processing
Bone marrow processing
Fresh iliac crest bone marrow aspirates from healthy human donors were collected at the Perelman School of Medicine, University of Pennsylvania, in heparinized syringes and transported to the lab for further processing within 4 hours of sample collection. Cells were isolated from bone marrow aspirate samples by placing sample on top of a 100um cell strainer filter (Corning) followed by mechanical dissociation using the flat end of a 5ml syringe (BD Biosciences). Cells were washed with DPBS and then underlaid with room temperature 96% Ficoll (GE Healthcare) prior to centrifugation at 400 x g for 30 minutes with 3 acceleration and no brake. Following centrifugation, mononuclear cells were harvested and washed with DPBS before RBC lysis was performed in ACK Lysis Buffer (Gibco). Cells were resuspended in cRPMI prior to cell counting. All samples were processed and stored at room temperature unless otherwise noted.

Flow cytometry
Sample Staining
Single cell suspensions isolated from rhesus macaque, human, or mouse tissues were prepared for flow cytometry by first incubating the cells with the appropriate Fc block (rhesus/human: Human TruStain FcX, Biolegend or mouse: purified rat anti-mouse CD16/CD32, BD Biosciences) in combination with a viability dye (Live/Dead Aqua or Violet, ThermoFisher Scientific). Following the Fc block and viability staining step, panels of fluorescently-conjugated antibodies were used to stain markers of interest. Details on markers targeted, antibody clones used, and fluorescent conjugates used can be found in Supplementary Table 1. All cells were stained in a 1:1 mix of FACS buffer (1% FBS 2mM EDTA in PBS) and Brilliant Violet Stain Buffer (BD Biosciences). Staining for human and rhesus samples was conducted at room temperature. Staining for mouse samples was conducted at 4C.

Instrument	Symphony A5 (BD Biosciences), Sony Cell Sorter SH800
Software	FACSDiva (v ,BD Biosciences), Sony Cell Sorter Software (v2.1.5, Sony), Flowjo (v10.6.2, BD Biosciences)
Cell population abundance	Samples were sorted as 3 drop single cell purity and purity of sorted populations was determined by reanalysis of sorted populations on cell sorting instrument. Sort purities are reported for NHP and human sorting experiments are reported in Supplemental Figures 6 and 7.
Gating strategy	Gating strategies for flow cytometry experiments are described in materials and methods or within the text of the manuscript. Gating strategies for sorting experiments are shown in Supplemental Figures 6 and 7.

☒ Tick this box to confirm that a figure exemplifying the gating strategy is provided in the Supplementary Information.
